# Bioinformatics analysis identifies coagulation factor II receptor as a potential biomarker in stomach adenocarcinoma

**DOI:** 10.1038/s41598-024-52397-6

**Published:** 2024-01-30

**Authors:** Xingwei Wu, Shengnan Wang, Chenci Wang, Chengwei Wu, Zhiyong Zhao

**Affiliations:** 1 Department of Thyroid and Breast Surgery, The Second Affiliated Hospital of Wannan Medical College, Wuhu, 241000 Anhui China; 2 Clinical Laboratory, Traditional Chinese Hospital of Lu’an, Anhui University of Chinese Medicine, Lu’an, 237000 Anhui China; 3 Department of Pathology, Fuyang People’s Hospital, Anhui Medical University, Fuyang, 236000 Anhui China; 4 Department of Oncology, Funan County People’s Hospital, Fuyang, 236000 Anhui China; 5 Department of Critical Care Medicine, The Second Hospital Affiliated to Jiaxing College, Jiaxing, 314000 Zhejiang China

**Keywords:** Computational biology and bioinformatics, Molecular biology, Biomarkers

## Abstract

Coagulation factor 2 thrombin receptor (*F2R*), a member of the G protein-coupled receptor family, plays an important role in regulating blood clotting through protein hydrolytic cleavage mediated receptor activation. However, the underlying biological mechanisms by which *F2R* affects the development of gastric adenocarcinoma are not fully understood. This study aimed to systematically analyze the role of *F2R* in gastric adenocarcinoma. Stomach adenocarcinoma (STAD)-related gene microarray data and corresponding clinicopathological information were downloaded from the Cancer Genome Atlas (TCGA) and Gene Expression Omnibus (GEO) databases. Differential expression genes (DEGs) associated with *F2R* were analyzed using Gene Ontology (GO), Kyoto Encyclopedia of Genes and Genomes (KEGG), gene set enrichment analysis (GSEA), and protein–protein interaction (PPI) networks. *F2R* mRNA expression data were utilized to estimate stromal cell and immune cell scores in gastric cancer tissue samples, including stromal score, immune score, and ESTIMATE score, derived from single-sample enrichment studies. Analysis of TCGA and GEO databases revealed significantly higher *F2R* expression in STAD tissues compared to normal tissues. Patients with high *F2R* expression had shorter survival times than those with low *F2R* expression. *F2R* expression was significantly correlated with tumor (T) stage, node (N) stage, histological grade and pathological stage. Enrichment analysis of *F2R*-related genes showed that GO terms were mainly related to circulation-mediated human immune response, immunoglobulin, cell recognition and phagocytosis. KEGG analysis indicated associations to extracellular matrix (ECM) receptor interactions, neuroactive ligand-receptor interactions, the phosphoinositide-3-kinase-protein kinase B/Akt (PI3K-AKT) signaling pathway, the Wnt signaling pathway and the transforming growth factor-beta (TGF-β) signaling pathway. GSEA revealed connections to DNA replication, the Janus kinase/signal transducers and activators of transcription (JAK-STAT) signaling pathway, the mitogen-activated protein kinase (MAPK) signaling pathway and oxidative phosphorylation. Drug sensitivity analysis demonstrated positive correlations between *F2R* and several drugs, including BEZ235, CGP-60474, Dasatinib, HG-6-64-1, Aazopanib, Rapamycin, Sunitinib and TGX221, while negative correlation with CP724714, FH535, GSK1904529A, JNK-9L, LY317615, pyrimidine, rTRAIL and Vinorelbine. Knocking down *F2R* in GC cell lines resulted in slowed proliferation, migration, and invasion. All statistical analyses were performed using R software (version 4.2.1) and GraphPad Prism 9.0. p < 0.05 was considered statistically significant. In conclusion, this study underscores the significance of *F2R* as a potential biomarker in gastric adenocarcinoma, shedding light on its molecular mechanisms in tumorigenesis. *F2R* holds promise for aiding in the diagnosis, prognosis, and targeted therapy of STAD.

## Introduction

Gastric cancer (GC) ranks as one of the most common human cancers globally, standing fifth in terms of incidence and representing the fifth leading cause of cancer-related deaths^1^. Notably, in East Asia, including China, the mortality rate is high^2–4^. Due to the lack of apparent symptoms in the early stages of GC, many patients are diagnosed at advanced stage^5,6^. STAD is the most common and malignant subtype^7^. Surgery remains the cornerstone of GC treatment. Despite recent advances in diagnosing and treating STAD, the prognosis for patients remains poor due to tumor recurrence and distant metastasis^8^. STAD is a multifactorial outcome, with complex interactions between *Helicobacter pylori* (*H. pylori*) and host vulnerabilities playing a dominant role in its development^9^. Like most cancers, complex interactions between multiple pro- and anticancer signaling pathways control the development and progression of GC^10–12^. Therefore, it is essential to study tumor-associated signaling pathways^13^. Understanding the molecular mechanisms of STAD tumorigenesis is imperative for identifying prognostic markers and new practical therapeutic approaches^14^. In recent years, mining central genes related to STAD from RNA-seq data in the TCGA database has become a prominent area of research. By analyzing the central gene of STAD, investigating the competing endogenous RNA (ceRNA) network significantly related to the prognosis of GC, and assessing the sensitivity of immune drugs, leveraging the TCGA database holds promise for advancing the diagnosis, prognosis and targeted therapy of STAD.

*F2R*, also known as protease-activated receptor (*PAR-1*), belongs to the PAR family. The PAR protein family is an evolutionary conserved regulator of polarity found asymmetrically in polarized cells. Cross-regulatory interactions between PAR proteins and other conserved polarity complexes establish a stable asymmetry within cells. These interactions also shape the evolution of PAR protein distribution in response to cues such as cytoskeleton transport^15,16^. Thrombin, matrix metalloproteinase 1 (MMP1), coagulation factor VII, kinase-releasing enzyme-related peptidase six (KLK6), and MMP2 act as key ligands, binding to *F2R* and triggering its signaling pathways^17,18^. Previous studies have reported elevated expression of *F2R* in various cancers such as lung adenocarcinoma^19,20^, glioma^21,22^, melanoma^23^, and breast cancer^24,25^. In these cases, elevated *F2R* levels are closely associated with malignant biological behaviors, including tumor invasion, proliferation, and angiogenesis. However, research on *F2R* in STAD remains limited. This study aimed to investigate the prognostic value and potential mechanisms of *F2R* in STAD.

Nearly 98% of the human transcriptome consists of noncoding RNA (ncRNA), specifically long-stranded noncoding RNAs (lncRNAs) defined as those exceeding 200 nucleotides^26^. These molecules exhibit limited or absent protein-coding capacity due to the lack of the open reading frame (ORF). However, they exert regulatory control over gene expression through various mechanisms, including epigenetic modifications, nuclear translocation, gene transcription, and mRNA translation^27^. A growing number of studies have shown that noncoding RNAs play a crucial role in regulating the biological functions of various tumors, including GC. In many tumors, lncRNAs function as ceRNAs, which compete with microRNAs (miRNAs) to upregulate the expression of target genes. In this intricate mechanism, lncRNAs can alleviate the repression imposed by miRNAs on their target genes, serving as miRNA sponges in many tumors and assuming the role of ceRNAs^28–30^. Specific examples include the lncRNA LINC01270/miR-326/EFNA3 axis, which exacerbates GC progression^31^. Overexpression of HOXC-AS1 acts as a sponge for miR-99a-3p, leading to the upregulation of MMP8 and ultimately promoting the GC progression^32^. The KCNQ1OT1/miR-378a-3p/RBMS1 axis is an important prognostic factor and therapeutic target for GC^33^. While these studies suggest the involvement of ceRNA networks in the progression of GC, further investigation is needed to understand the ceRNA networks that are significantly associated with the prognosis of GC.

Immunotherapeutic approaches, such as immune checkpoint blockade (ICB) and chimeric antigen receptor T cells (CAR-T), have made significant progress in recent years and revolutionized treatment options. Monoclonal antibody therapy targeting immune checkpoint proteins PD-1/PD-L1 and CTLA-4 have rapidly progressed, showing impressive response rate in various tumors, including RCC, melanoma, lung cancer, GC, and other tumors^34^. The potential of immunotherapy in GC patients has attracted increasing attention^35^. While ICB therapy have demonstrated durable disease control in certain tumor patients, the implementation of multitarget or targeted multicellular therapies, as well as combinations with other treatment modalities, may improve antitumour efficacy. Therefore, efforts toward antitumor therapies targeting immune cells still have a considerable journey ahead to achieve synergistic effects^36^. Targeted therapy is a fundamental strategy in the clinical treatment of cancer. Identifying potential antitumour agents that could benefit tumor treatment allows for the analysis of response among tumor patients in different risk subgroups to antitumour drugs, offering novel insights for precision targeted therapy for tumor patients^37^.

Bioinformatic analysis of sequencing data from the TCGA database enables the identification of gene regulatory pathways and disease networks. In this study, we compared differentially expressed genes in patients using STAD samples from the TCGA database. The expression profiles of these genes were correlated with patients’ clinical data. The RNA expression analysis data included 375 tumor samples and 32 nontumor samples. To further explore the potential biological functions of *F2R* in STAD, bioinformatics tools were employed to validate molecular mechanisms through GSEA and GO/KEGG enrichment analysis. We systematically screened miRNAs and lncRNAs upstream of *F2R*, constructing a ceRNA network. This network was investigated for its potential regulatory role in STAD development. In this study, we investigated the prognostic value of *F2R* in GC and comprehensively evaluated its potential value. Our findings contribute to the identification of novel prognostic biomarkers and provide insights into potential molecular mechanisms that influence GC prognosis. Finally, in vitro and in vivo experiments were performed to validate the expression and function of *F2R* in GC.

## Results

### *F2R* expression in STAD

**Figure 1:**
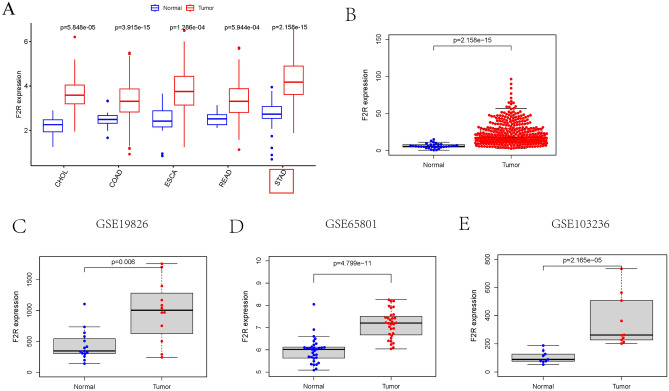
The expression level of *F2R* in STAD samples was significantly increased. (**A**) The expression of *F2R* in different tumor tissues in the UCSC-xena database, and the expression of *F2R* in STAD increased. (**B**) The expression of *F2R* in STAD based on the TCGA data set increased. (**C–E**) Elevated *F2R* expression in STAD based on GEO datasets GSE19826, GSE65801 and GSE103236.

Our dataset, comprising 375 patients with STAD, was obtained from TCGA and included both clinical information and gene expression data. Various clinical characteristics, such as gender, age, histological grade, pathological grade, T stage, N stage, M stage and vital status, were collected (Table S1). The Perl programming language was used to match gene expression data with clinical information, eliminating samples of unknown or incomplete clinical data. The patient data were categorized into high and low *F2R* expression groups based on the median level of *F2R* gene expression. Table 1 provides a comprehensive overview of the differences in clinical data of GC patients in different *F2R* expression groups. Furthermore, the analysis of *F2R* expression across different tumor types using the UCSC Xena database revealed increased *F2R* expression in various tumors, including cholangiocarcinoma (CHOL), colon adenocarcinoma (COAD), esophageal carcinoma (ESCA), rectum adenocarcinoma (READ), and STAD (Fig. 1A). Notably, based on TCGA data, *F2R* expression was significantly higher in STAD tissues compared to normal tissues (p = 2.158e−15) (Fig. 1B). Consistent with these findings, analysis of three GEO datasets demonstrated higher *F2R* expression in the STAD group (Fig. 1C–E).

**Table 1:** STAD patient characteristics in the TCGA database.

Characteristic	Low expression of F2R	High expression of F2R	p
n	188	187	
T-stage, n (%)			**0.038**
T1	14 (3.81%)	5 (1.36%)	
T2	42 (11.44%)	38 (10.35%)	
T3	87 (23.71%)	81 (22.07%)	
T4	43 (12.11%)	57 (15.53%)	
N-stage, n (%)			**0.048**
N0	63 (17.65%)	48 (13.45%)	
N1	48 (13.45%)	49 (13.73%)	
N2	36 (10.08%)	39 (10.92%)	
N3	31 (8.68%)	43 (12.04%)	
M-stage, n (%)			0.847
M0	165 (46.48%)	165 (46.48%)	
M1	13 (3.67%)	12 (3.38%)	
Pathologic stage, n (%)			0.076
I	34 (9.58%)	19 (5.35%)	
II	57 (16.06%)	54 (15.21%)	
III	65 (18.31%)	85 (23.94%)	
IV	22 (6.20%)	19 (5.35%)	
Histological grade, n (%)			**0.006**
G1	5 (1.37%)	5 (1.37%)	
G2	82 (22.40%)	55 (15.03%)	
G3	96 (26.23%)	123 (33.61%)	
Gender, n (%)			0.969
Female	67 (17.87%)	67 (17.87%)	
Male	121 (32.27%)	120 (32.00%)	
Age, n (%)			0.81
≤ 70	120 (32.35%)	119 (32.08%)	
> 70	68 (18.33%)	64 (17.25%)	
Tumour status (%)			0.324
With tumor	43 (13.35%)	34 (10.56%)	
Tumor free	121 (37.58%)	124 (39.43%)	
Residual tumour, n (%)			0.9
R0	152 (46.20%)	146 (44.38%)	
R1	6 (1.82%)	9 (2.74%)	
R2	10 (3.04%)	6 (1.82%)	
OS event, n (%)			0.381
Live	147 (39.20%)	153 (40.80%)	
Death	41 (10.93%)	34 (9.07%)	

Significant values are in bold.

### Association between *F2R* gene expression and clinical characteristics in STAD patients

**Figure 2:**
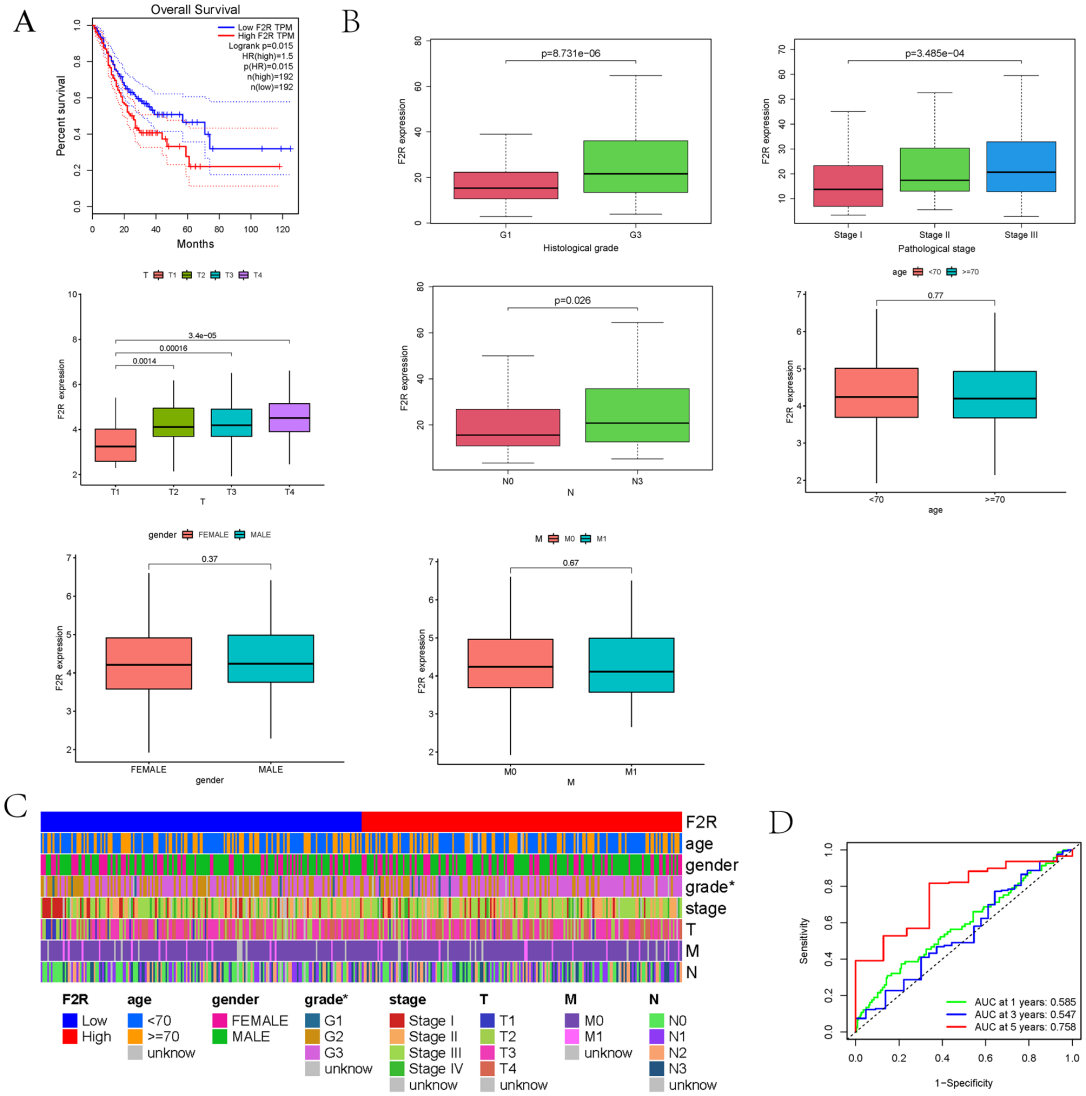
The expression of *F2R* is related to the prognosis and clinicopathological characteristics of patients with STAD. (**A**) Based on the TCGA data set, the total survival time of STAD patients with high *F2R* expression was significantly shorter than that of STAD patients with low *F2R* expression. (**B**) *F2R* expression was positively correlated with the clinical Histological grade, Pathological stage, T stage and N stage, but not with age, gender and M stage. (**C**) Clinical-related thermal imaging analysis in patients with STAD with high and low expression of *F2R*. (**D**) The survival work characteristic curve of patients with STAD showed that *F2R* had a good predictive value for 5-year survival (AUC = 0.758).

Kaplan–Meier survival curves revealed that patients with elevated *F2R* expression exhibited shorter survival times compared to those with low *F2R* expression (p = 0.029) (Fig. 2A). The relationship between *F2R* gene expression and clinicopathological variables in GC patients was further explored using the Wilcoxon signed-rank test and logistic regression analysis. *F2R* mRNA expression was examined across patient groups categorized by age, gender, clinical grade, T stage, N stage, and M stage of the patients. The findings indicated a significant association between *F2R* overexpression and T stage (T2, T3, T4, and T1; p = 0.0014, 0.00016, 3.4e−05), N stage (N0 and N3; p = 0.026), histological grade (G1 and G3; p = 8.731e−06), and pathological stage (stage I and stage III; p = 3.485e−04). Notably, *F2R* gene expression was independent of clinical factors, including age, sex, and M stage (Fig. 2B). Patients with GC were stratified into two groups based on *F2R* expression levels, and clinically relevant thermography was employed to analyze patients’ variables and the high/low expression of *F2R*. The results revealed a statistically significant correlation between patients’ grade stage and *F2R* expression (p < 0.05) (Fig. 2C). The diagnostic value of *F2R* mRNA expression for predicting patient survival at 1, 3, and 5 years was assessed using ROC curves, yielding areas under the curve (AUCs) of 0.585, 0.547, and 0.758, respectively (Fig. 2D). Furthermore, univariate logistic regression analysis explored the relationship between *F2R* gene expression and the clinicopathological characteristics in GC patients. The results highlighted a significant association between high *F2R* gene expression and histological grade, pathological stage, T stage, and race. However, no significant differences were observed between *F2R* gene expression and other clinicopathological variables, including age, sex, N stage, M stage, tumor status and residual tumor (Table 2).

**Table 2:** F2R expression correlated with clinical pathological characteristics (logistic regression).

Characteristics	Total (N)	Odds ratio (OR)	p value
Age (> 60 vs. ≤ 60 years)	371	0.720 (0.465–1.113)	0.141
Gender (female vs male)	375	0.992 (0.650–1.514)	0.969
Histological grade (G1 & G2 vs G3 & G4)	366	1.858 (1.2189–2.846)	**0.004**
Pathological stage (stage II & stage III & stage IV vs stage I)	352	1.979 (1.091–3.684)	**0.027**
T stage (T2 & T3 & T4 vs T1)	367	2.932 (1.096–9.231)	**0.043**
M stage (M1 & M0)	355	0.923 (0.404–2.094)	0.847
N stage (N1 & N2 & N3 vs N0)	357	1.471 (0.938–2.317)	0.094
Race (White & Black or African American & Native Hawaiian or Other Pacific Islander vs Asian)	381	1.898 (1.122–3.258)	**0.018**
Tumour status (with tumour vs tumour free)	381	0.735 (0.437–1.227)	0.241
Residual tumour (R1 & R2 vs R0)	387	0.938 (0.443–1.973)	0.864

Categorical dependent variable, greater or less than the median expression level.

*T* topography distribution, *N* lymph node metastasis, *M* distant metastasis.

Significant values are in bold.

### Development of a prognostic model integrating *F2R* expression and clinical factors

**Figure 3:**
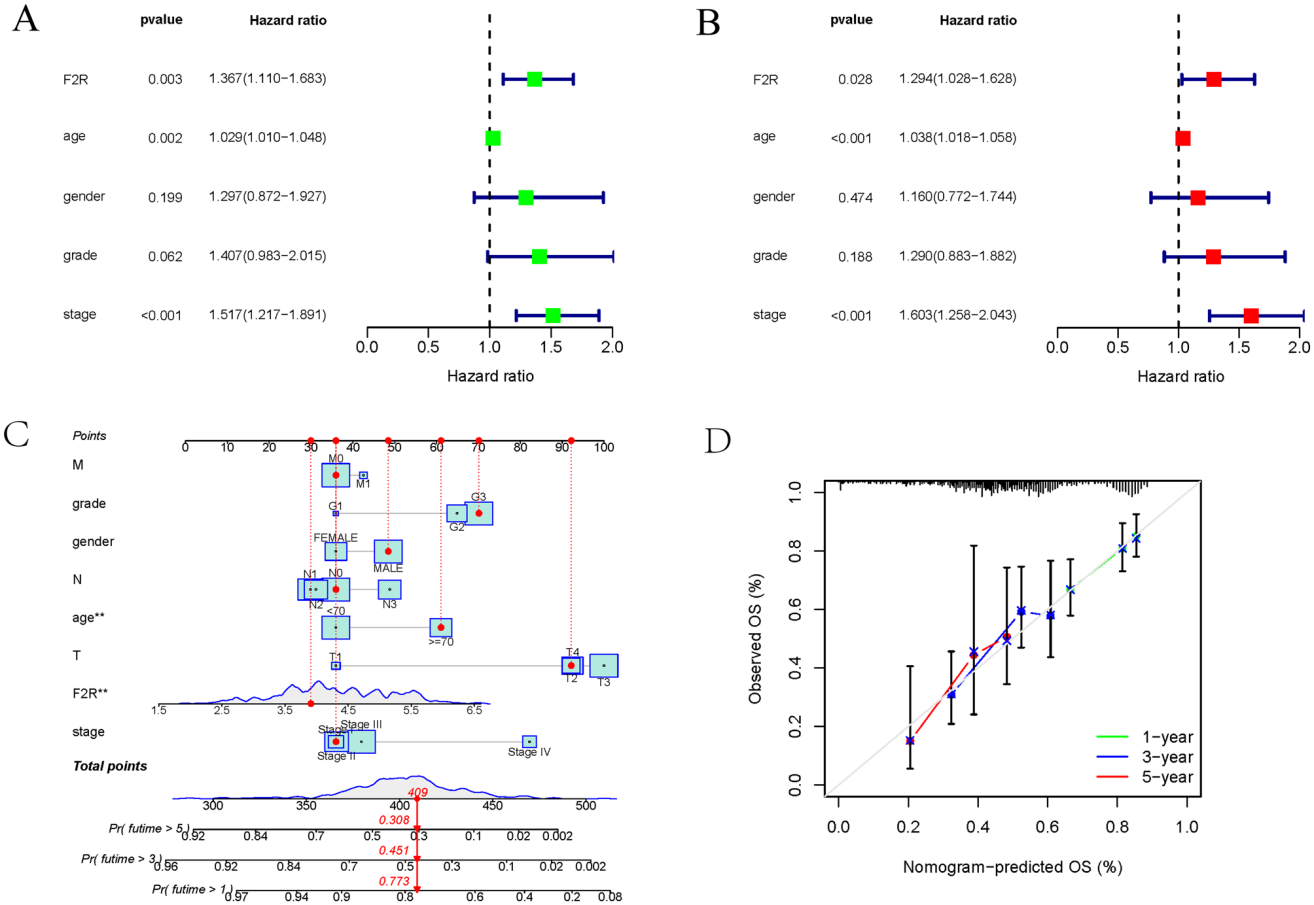
Clinical significance of *F2R* gene expression in the prognosis of STAD. (**A**) Univariate analysis showed that the expression levels of stage, age and *F2R* were independent predictors of survival time in patients with STAD. (**B**) Multivariate analysis showed that *F2R* expression was a reliable prognostic factor for patients with STAD. (**C**) A nomogram integrating *F2R* and other prognostic factors of STAD based on TCGA data. (**D**) The calibration curve of the nomogram.

Univariate regression analysis unveiled that *F2R* gene expression was a high-risk factor for GC (HR 1.367; confidence interval, 1.110–1.683; p = 0.003) (Fig. 3A). In the subsequent multivariate analysis, *F2R* mRNA expression emerged as an independent risk factor for overall survival in GC patients (HR 1.294; confidence interval, 1.028–1.628; p = 0.028) (Fig. 3B). Both univariate and multifactorial Cox regression analyses consistently identified *F2R* gene expression as a high-risk factor for STAD (Table 3). Utilizing *F2R* expression and other clinical parameters, we developed a prediction model for overall survival prediction, integrating *F2R* as a biomarker for GC. A nomogram was established to visually represent this prediction model (Fig. 3C). A calibration curve evaluated the performance of the nomogram for *F2R* expression versus 1-year, 3-year, and 5-year survivals (Fig. 3D).

**Table 3:** Univariate analysis and multivariate analyses of STAD patients overall survival in the TCGA database.

Characterical	Total (N)	Univariate analysis		Multivariate analysis	
		Hazard ratio (95% Cl)	p value	Hazard ratio (95% Cl)	p value
T stage (T2 & T3 & T4 vs. T1)	367	7.248 (1.011–51.980)	**0.049**	3.804 (0.483–29.971)	0.205
N stage (N1 & N2 & N3 vs. N0)	357	1.507 (0.965–2.354)	0.071	1.198 (0.7–2.05)	0.511
M stage (M1 vs. M0)	355	2.048 (1.096–3.827)	**0.025**	2.976 (1.541–5.747)	**0.001**
Pathological stage (stage II & stage III & stage IV vs. stage I)	352	2.111 (1.062–4.195)	**0.033**	1.182 (0.502–2.781)	0.702
Age (> 60 vs. ≤ 60 years)	371	1.029 (1.01–1.048)	**0.002**	1.038 (1.018–1.058)	**0**
Gender (male vs. female)	375	1.297 (0.872–1.927)	0.199	1.160 (0.772–1.744)	0.474
Histological grade (G2 & G3 vs. G1)	366	2.894 (0.404–20.76)	0.29	2.276 (0.314–16.480)	0.415
F2R (high vs. low)	375	1.367 (1.110–1.683)	**0.003**	1.294 (1.028–1.628)	**0.028**

*T* topography distribution, *N* lymph node metastasis, *M* distant metastasis.

Significant values are in bold.

### Construction of the lncRNA–miRNA–mRNA ceRNA regulatory network

**Figure 4:**
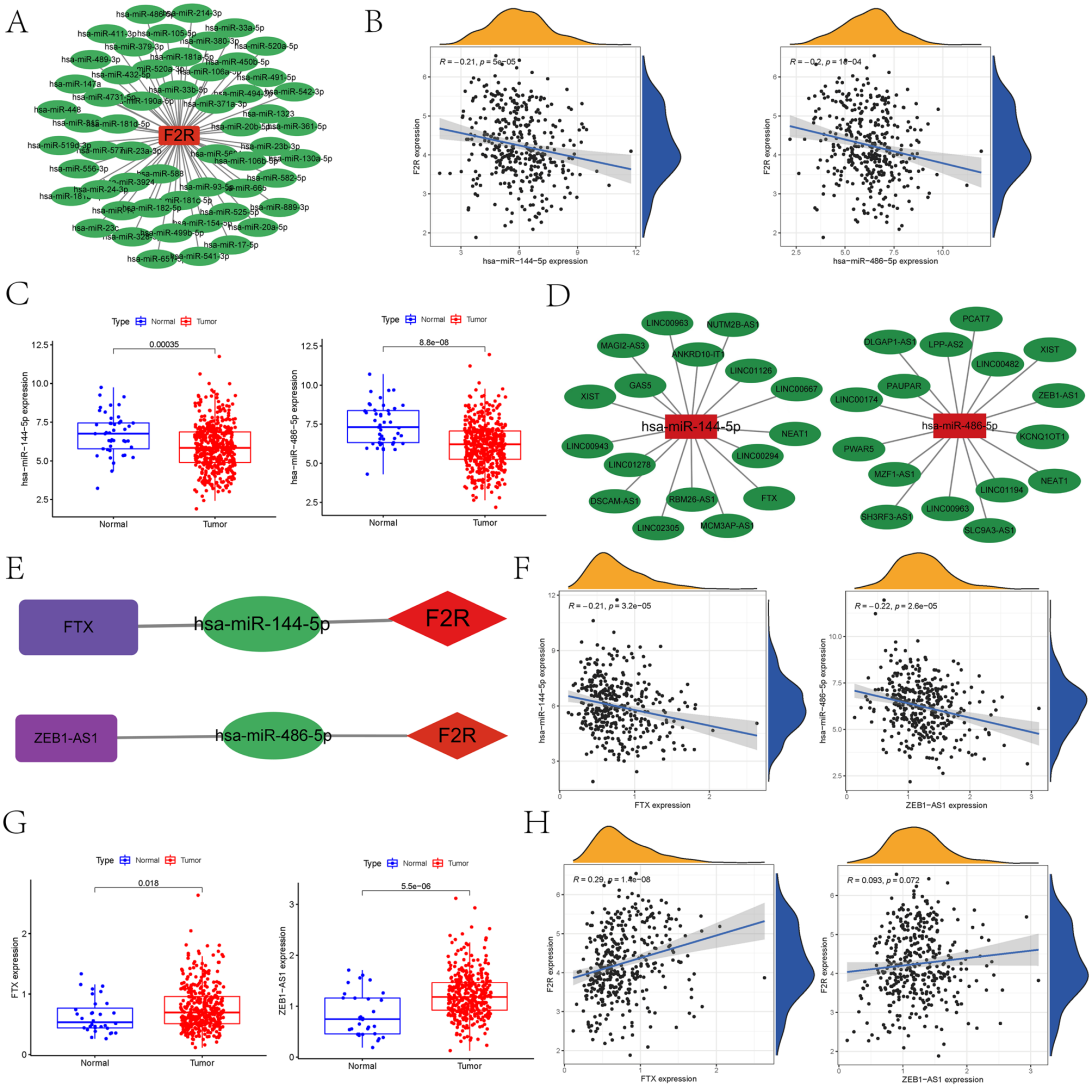
Screening the upstream miRNA and lncRNA of *F2R* and constructing the ceRNA network diagram. (**A**) Network mapping of *F2R*-related miRNAs based on the Starbase database. (**B**) The expression of *F2R* based on the TCGA dataset was negatively correlated with the expression of *hsa-miR-144-5p* and *hsa-miR-486-5p*. (**C**) The expression of *hsa-miR-144-5p* and *hsa-miR-486-5p* in STAD tissues based on the TCGA data set decreased. (**D**) Mapping of *hsa-miR-144-5p* and *hsa-miR-486-5p* related lncRNA networks based on the Starbase database. (**E**) To construct an *F2R*-centered ceRNA network. (**F**) Based on the TCGA data set, the expression of *hsa-miR-144-5p* was negatively correlated with the expression of *FTX*, and the expression of has-miR-486-5p was negatively correlated with the expression of *ZEB1-AS1.* (**G**) The expression of *FTX* and *ZEB1-AS1* in STAD based on the TCGA data set increased. (**H**) Based on the TCGA data set, *F2R* expression was positively correlated with *FTX* and *ZEB1-AS1* expression.

Following the ceRNA hypothesis, where miRNAs negatively modulate gene expression by targeting mRNA, and lncRNAs can indirectly regulate this process by binding to these miRNAs, we aimed to establish a ceRNA network targeting *F2R* using bioinformatic methods. Using miRNA target prediction screening in the starBase database, 56 miRNAs associated with *F2R* were identified (Fig. 4A). A correlation table between *F2R* and these miRNAs is provided in Table S2, revealing strong negative correlations for *hsa-miR-144-5p* and *hsa-miR-486-5p*. These two miRNAs were chosen for further investigation due to their negative correlation with *F2R* expression and lower expression in GC tissues compared to normal tissues (Fig. 4B,C). Further details on the expression of other miRNAs in STAD and their correlation with *F2R* are presented in Supplementary Fig. S1A,B. To identify lncRNAs potentially interacting with hsa-miR-144-5p and hsa-miR-486-5p, the starBase database was utilized for further analysis. The detailed correlations between these miRNAs and lincRNA are presented in Tables S3 and S4. Based on these findings, a ceRNA regulatory network was constructed using Cytoscape software (Fig. 4D,E). We identified upstream lncRNAs that exhibited a negative correlation with the expression of *hsa-miR-144-5p* and *hsa-miR-486-5p*, while demonstrating a positive correlation with *F2R* expression. Specifically, *lncRNA-FTX* was found to regulate *F2R* expression in GC cells through the *hsa-miR-144-5p/F2R* axis, while *lncZEB1-AS1* regulated *F2R* expression through the *hsa-miR-486-5p/F2R* axis, thereby modulating *F2R* expression in GC cells. Moreover, the expression levels of lncRNA *FTX* and *lncZEB1-AS1* were significantly elevated in GC tissues compared to adjacent normal tissues (Fig. 4F–H). This upregulation suggests that these lncRNAs may be potential biomarkers for GC diagnosis or prognosis.

### Enrichment analyses of *F2R*-related through GSEA, GO, and KEGG

**Figure 5:**
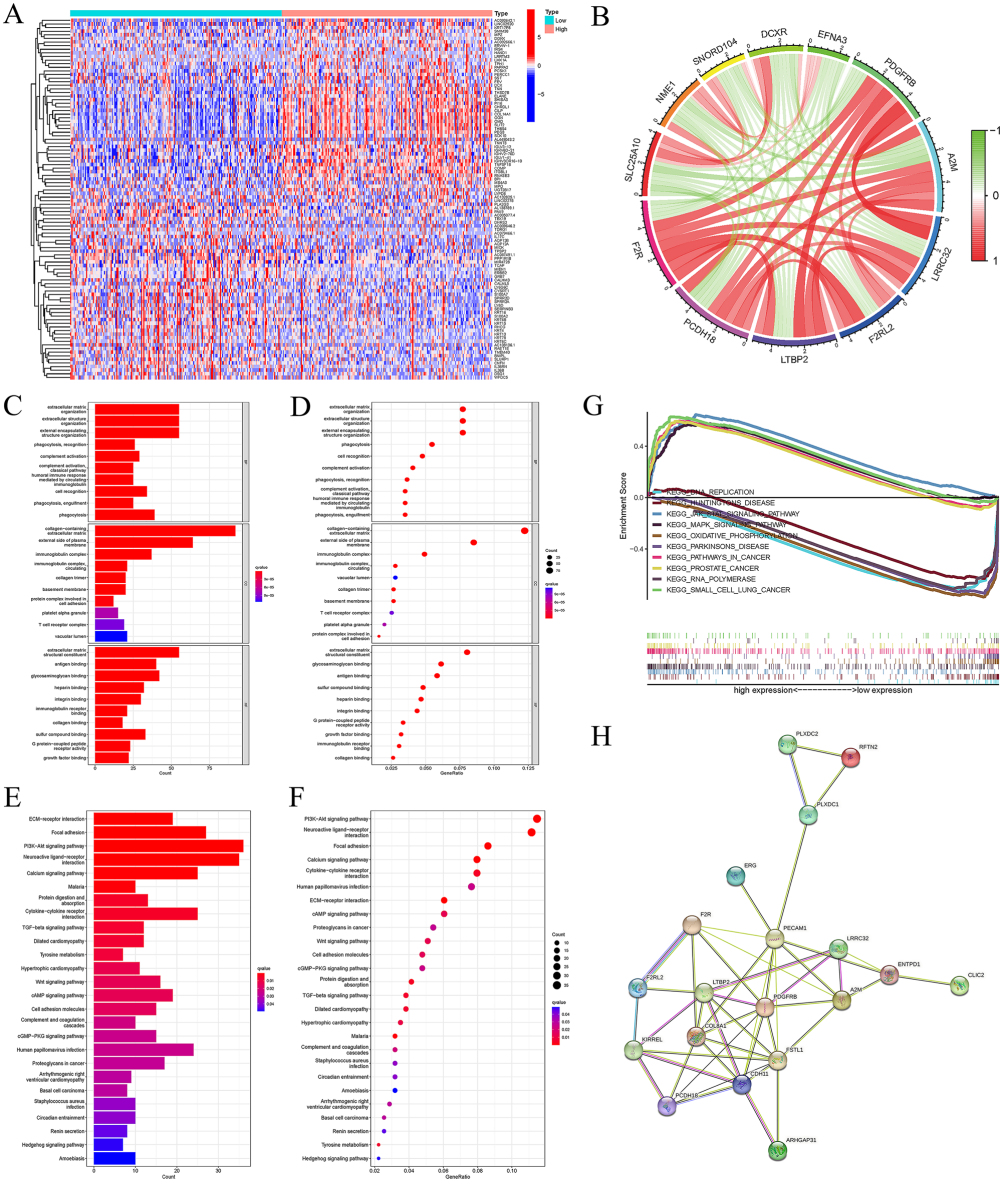
Gene enrichment analysis to identify *F2R*-related pathways. (**A**) Screening genes that are positively or negatively correlated with *F2R* expression to draw a gene heat map. (**B**) Analysis of 11 genes with significantly different expression related to *F2R*. (**C**–**D**) The enrichment analysis of gene ontology (GO) showed that *F2R*-related genes were mainly related to the completion of the activation of classical pathways, circulating mediated human immune response, immunoglobulin, cell recognition and phagocytosis. (**E**,**F**) The enrichment analysis of the Kyoto Encyclopedia of Genes and Genome (KEGG) showed that *F2R*-related genes mainly interact with ECM receptor, neuroactive ligand receptor, protein differentiation and uptake, cytokine-cyclin receptor interaction, PI3K-Akt signal pathway, cAMP signal pathway, Wnt signal pathway, cGMP PKG signal pathway and TGF-β Signal path correlation. (**G**) Gene cluster enrichment analysis (GSEA) showed that the signal pathway related to the expression of *F2R* mRNA in gastric cancer. The results showed that DNA replication, Huntington’s disease, JAK-STAT signal pathway, MAPK signal pathway and oxidative phosphorylation were related to the expression of *F2R*. (**H**) Protein–protein interaction (PPI) network was used to analyze genes related to *F2R*.

Genes highly associated with *F2R* were screened in GC using differential expression analysis. The identified DEGs were then classified into two groups based on high or low expression of *F2R* (Fig. 5A). Eleven genes with significant differential expression were further characterized. *PDGFRB*, *A2M*, *LRRC32*, *F2RL2*, *LTBP2*, and *PCDH18* exhibited a positive correlation with *F2R* expression. *EFNA3*, *DCXR*, *SNORD104*, *NME1*, and *SLC25A10* showed a negative correlation with *F2R* expression (Fig. 5B). To delve deep into the relationships between *F2R* and these genes, a comprehensive correlation analysis was conducted (Table S5). Supplementary Figure S2A–R provide detailed information on the correlation between *F2R* expression and these identified DEGs. To understand the functional relevance of the identified *F2R*-associated genes, GO and KEGG pathway analyses were conducted. GO analysis revealed that *F2R*-related genes were primarily enriched in functional categories related to immune response and cell communication (Fig. 5C–D). Notably, these genes showed significant enrichment for terms such as “completion of the activation of the classical pathway”, “circulating-mediated human immune response”, “immunoglobulins”, “and “cell recognition”, suggesting potential roles in antibody-mediated immunity and cell–cell interactions. KEGG pathway analysis provided further insights, highlighting enrichment in various signaling pathways crucial for cell–cell communication and regulation. Genes associated with *F2R* were significantly enriched in pathways such as ECM receptor interactions, neuroactive ligand-receptor interactions, protein differentiation and uptake, cytokine-cyclin receptor interactions, the PI3K-Akt signaling pathway, the cAMP signaling pathway, the Wnt signaling pathway, the cGMP-dependent protein kinase (cGMP/PKG) signaling pathway, and the TGF-β signaling pathway (Fig. 5E,F). To explore the potential regulatory mechanisms of the *F2R* gene in GC, GSEA was performed using data from the TCGA database. The results showed associations of *F2R* mRNA expression with DNA replication, Huntington’s disease, the JAK-STAT signaling pathway, the MAPK signaling pathway, and oxidative phosphorylation (Fig. 5G, Table S6). In addition, GSEA suggested possible involvement of *F2R* in the progression of GC and its association with the mammalian target of rapamycin (mTOR), p53 and T-cell receptor signaling pathways (Supplementary Fig. S3A–G). The PPI network also identified genes associated with *F2R* (Fig. 5H).

### Correlation between *F2R* and immune cell infiltration

**Figure 6:**
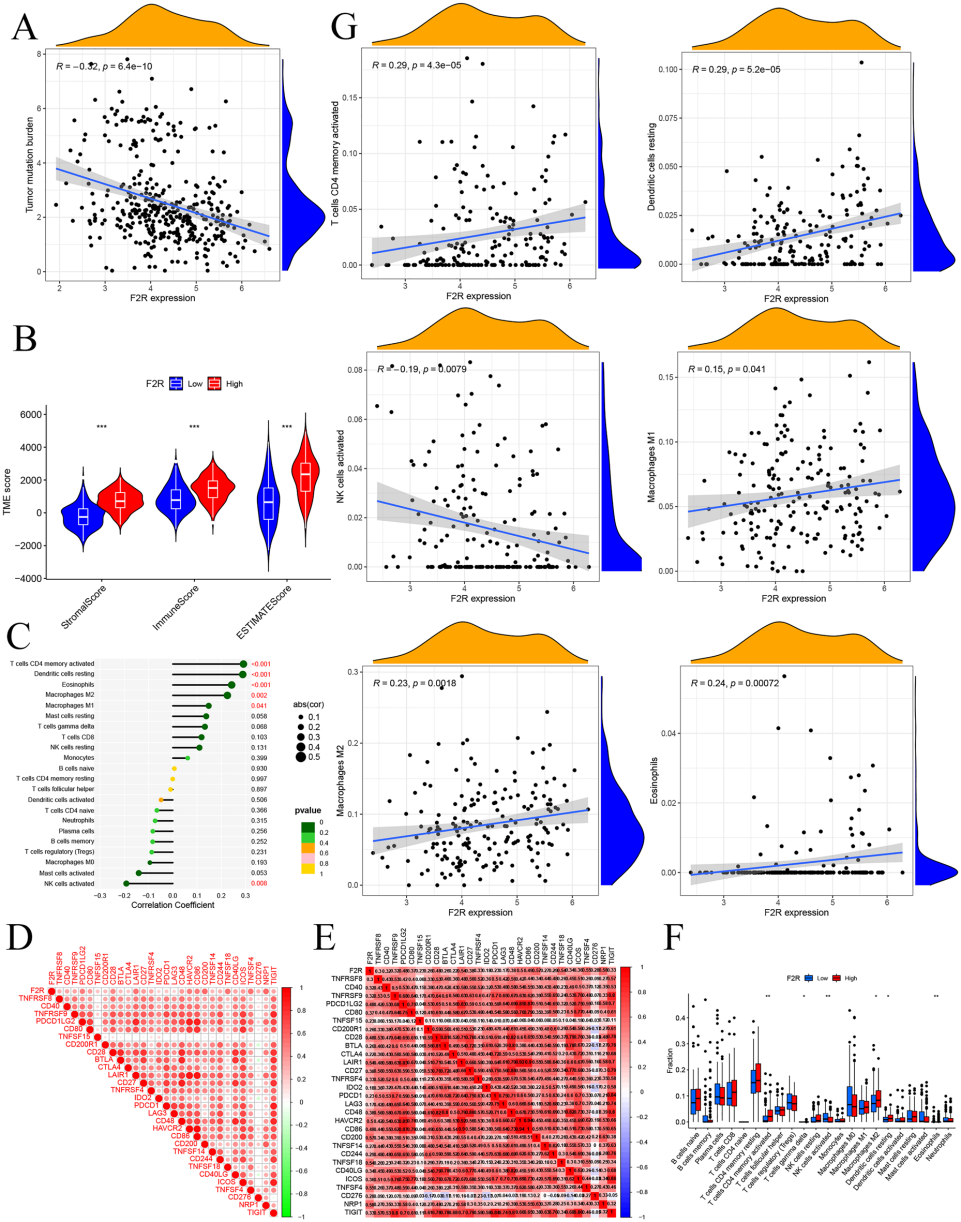
*F2R* may participate in the immune regulation and infiltration of cancer tissue. (**A**) The increase of *F2R* expression in STAD is related to the decrease of TMB. (**B**) The expression of *F2R* in STAD was divided into low expression group and high expression group, and the immune infiltration of Stromalscores and Immunocore in low and high-expression groups was analyzed. (**C**) CIBERSORT was used to calculate the correlation between *F2R* and 22 immune cells. (**D,E**) Analyze the correlation between *F2R* and immune-related genes. (**F**) Analyze the correlation between *F2R* and immune cells. (**G**) Analysis of the linear relationship between *F2R* and differential immune cells shows a positive regulatory relationship between *F2R* and immune cells.

Tumor mutational burden (TMB) plays a pivotal role in tumorigenesis and progression. We investigated the correlation between TMB and *F2R* expression in STAD and explored the connection between *F2R* expression and mutations in STAD. Our findings indicated a significant correlation between increased *F2R* expression in STAD and decreased TMB (Fig. 6A). Furthermore, STAD patients were stratified into low and high *F2R* expression groups based on their *F2R* expression levels. Analysis of stromal and immune cell infiltration in these groups revealed significant differences in StromalScore, Immunoscore, and estimated core scores, with higher scores observed in the high *F2R* expression group (Fig. 6B). Stromal score refers to the number of stromal cells in the tumor tissue, immune score refers to the number of immune cells in the tumor tissue, and estimate score refers to the purity of the tumor. To analyze the association between *F2R* and immune cell infiltration, we calculated the correlation between *F2R* and 22 immune cells using CIBERSORT. The results highlighted variations in the abundance of immune cell subtypes between low and high *F2R* expression groups. Notably, CD4 cell memory activation, dendritic cell quiescence, eosinophils, M2 macrophages, and M1 macrophages exhibited increased abundance in the *F2R* high expression group, while natural killer (NK) cells were activated in the *F2R* low expression group (Fig. 6C). Analysis of the linear relationship between *F2R* and immunological checkpoint genes showed that *F2R* had a positive regulatory relationship with immunological checkpoint genes (Fig. 6D,E). Further exploration of the correlation between *F2R* and immune-related genes demonstrated its involvement in the immune cell infiltration of GC. Specifically, CD4^+^ cell memory-activated, M1 macrophages, M2 macrophages, eosinophils, and resting dendritic cells showed positive correlations with *F2R*, whereas NK cell activation displayed a negative correlation (Fig. 6F,G). A comprehensive analysis of *F2R* and immune cell gene markers revealed a significant positive correlation between *F2R* and the expression of 21 immune response markers across various immune cell types (Table S7). A linear plot of the correlation between *F2R* and immune cell genes is shown in Supplementary Fig. S4. The collective evidence suggested that *F2R* plays a crucial role in immune regulation and infiltration within GC tissues.

### Relationship between *F2R* expression and immune cell infiltration in GC tissues

**Figure 7:**
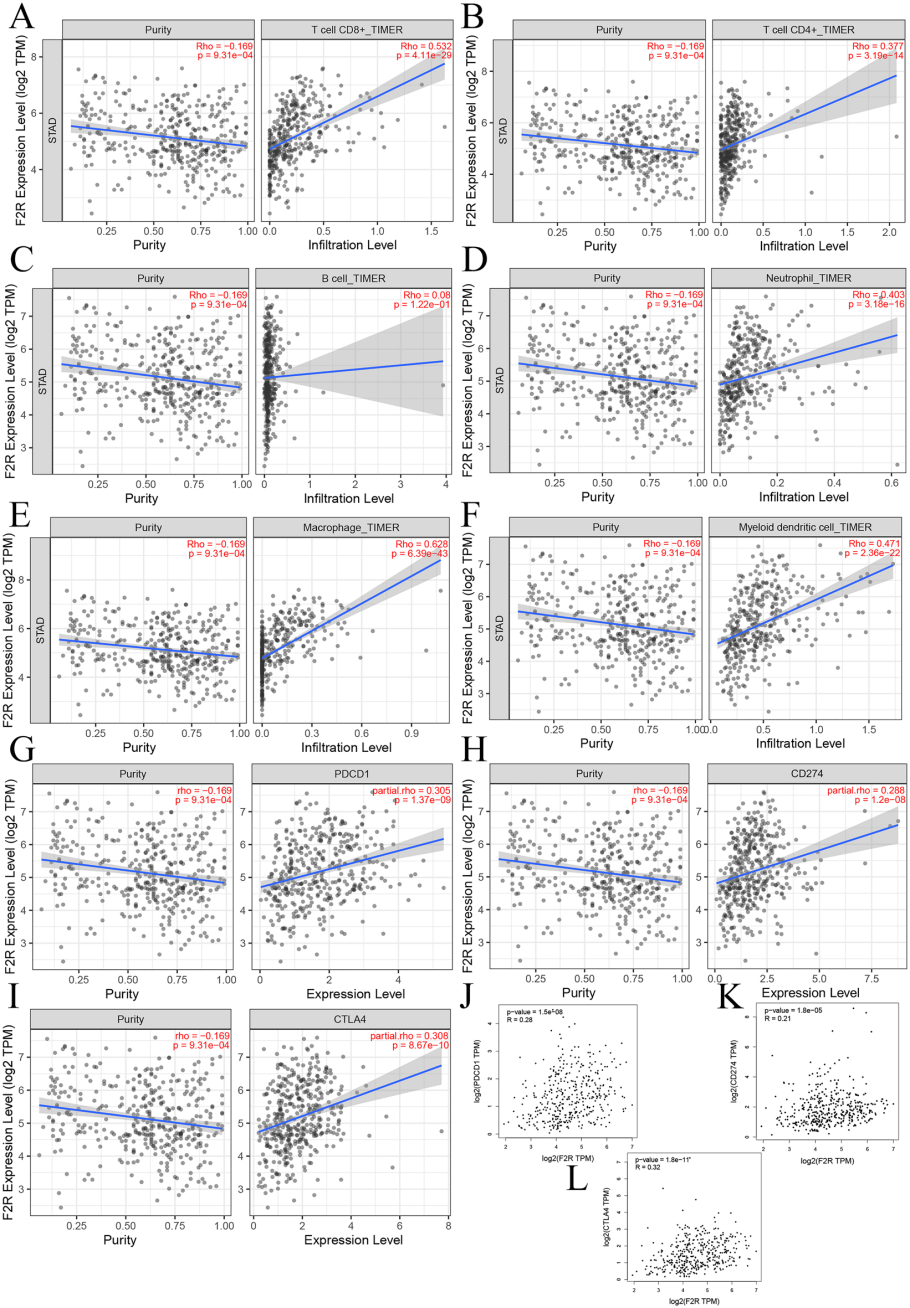
Correlation between immune cell infiltration, *PDCD1* (*PD1*), *CD274 (PD-L1)*, and expression of *CTLA4* and *F2R* in STAD. (**A–F**) TIMER database showed that the expression of *F2R* was significantly positively correlated with the infiltration of CD4 T cells, CD8 T cells, dendritic cell, macrophages, neutrophils and B cells. (**G–I**) TIMER database showed that the expression of *F2R* was positively correlated with the expression of CD274, PDCD1 and CTLA4. (**J–L**) Gepia database showed that the expression of *F2R* was positively correlated with the expression of *CD274*, *PDCD1* and *CTLA4*.

To substantiate the regulatory role of *F2R* in immune cell infiltration within GC tissues, our analysis of *F2R* and immune cell gene markers revealed a significant positive correlation with the expression of six immune response markers on B cells, CD4^+^ T cells, CD8^+^ T cells, M2 macrophages, neutrophils and dendritic cells (Fig. 7A–F). Additionally, we examined the association between *F2R* gene copy number and immune cells, and the results are shown in Supplementary Fig. S5A–E. The analysis consistently demonstrated a positive correlation between *F2R* and these immune cell types. Further investigating the relationship between *F2R* and immune checkpoints, we obtained consistent positive correlations between *F2R* expression and the immune checkpoints *CD274*, programmed cell death protein 1 (*PD1*), and cytotoxic T-lymphocyte associated protein 4 (*CTLA4*). These findings were validated by analyzing correlations using the TIMER and GEPIA databases (Fig. 7G–L). The relationship between *F2R* and other immune checkpoints is shown in Supplementary Fig. S6A,B.

### Correlation between *F2R* gene methylation status and prognosis in STAD patients

**Figure 8:**
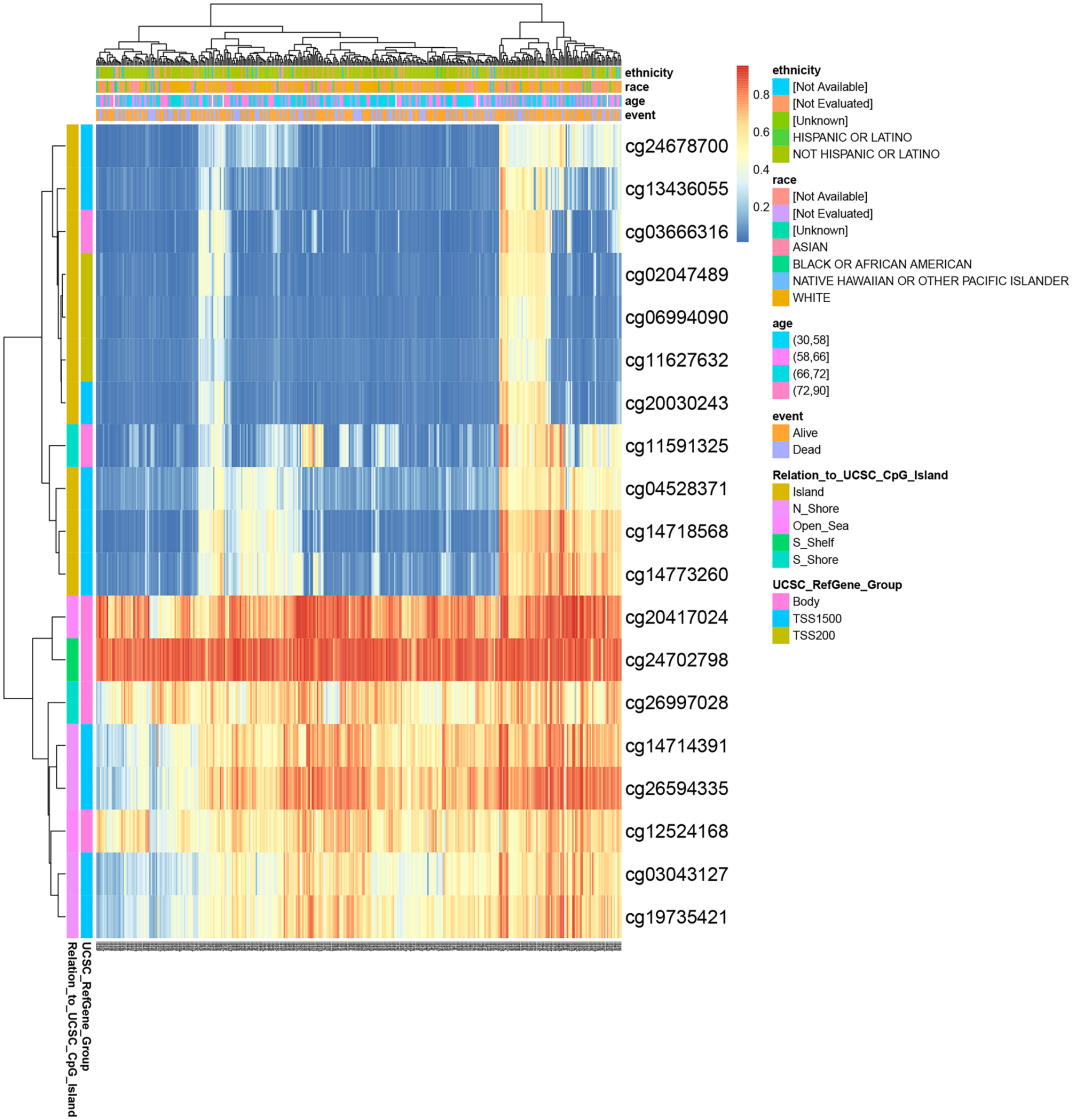
Methylation levels in the *F2R* gene are associated with the prognosis of STAD patients. The prognosis value of DNA methylation levels in the *F2R* gene and CpG islands in the *F2R* gene were analyzed using the MetSurv tool.

The prognostic significance of DNA methylation levels within the *F2R* gene and CpG islands was assessed using the MetSurv tool. The analysis identified 19 methylated CpG islands (cg20417024, cg24702798, cg26997028, cg14714391, cg26594335, cg12524168, cg03043127 and cg19735421) exhibiting elevated DNA methylation levels (Fig. 8). In addition, the methylation levels of 14 CpG islands (cg02047489, cg11627632, cg03666316, cg04528371, cg13436055, cg14773260, cg20030243, cg24678700, cg03043127, cg19735421, cg26594335, cg11591325, cg20417124, cg24702798) were found to be associated with prognosis of GC (Table S8). Of particular interest, elevated methylation levels in these 14 CpG islands, especially in cg03666316 and cg13436055, were correlated with poorer overall survival outcomes in STAD patients compared to those with lower CpG methylation levels of *F2R*.

### Correlation analysis between risk group and drug sensitivity

**Figure 9:**
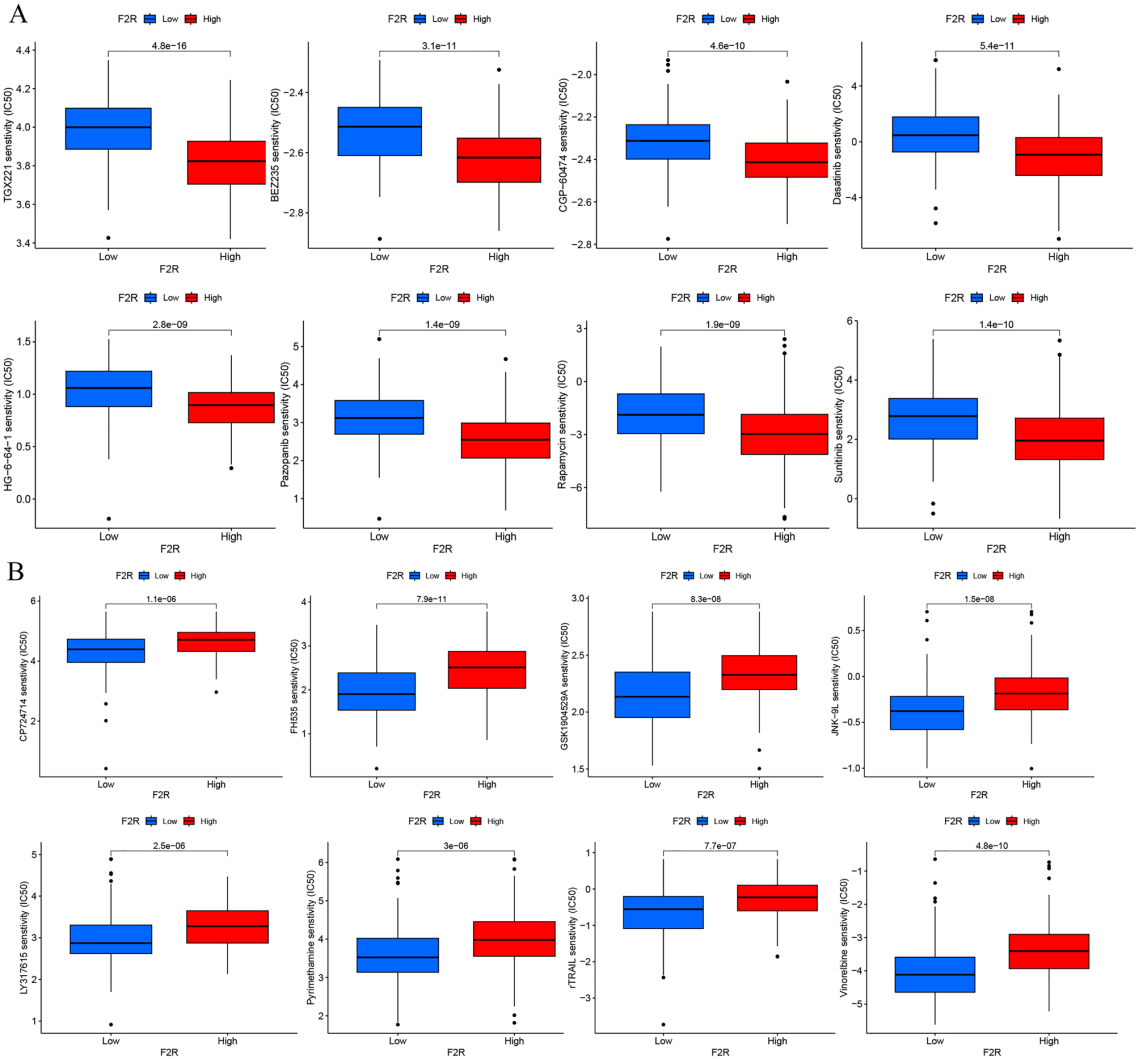
Predicted drug sensitivity in risk groups. (**A**) Sensitive drugs in high-risk groups. (**B**) Sensitive drugs in low-risk groups.

Genes associated with sensitivity and resistance to anticancer drugs have been extensively studied. In this investigation, we examined drug sensitivity within two risk groups, exploring the relationship between risk scores and IC_50_ values for various drugs potentially used to treat GC with high *F2R* expression. The prediction of the relationship between the *F2R* gene and chemical compound sensitivity was conducted using pRRophetic software. Our findings revealed that *F2R* was positively associated with BEZ235, CGP-60474, Dasatinib, HG-6-64-1, Pazopanib, Rapamycin, Sunitinib, and TGX221, while negatively associated with CP724714, FH535, GSK1904529A, JNK-9L LY317615, pyrimethamine, rTRAIL, and Vinorelbine (Fig. 9A,B).

**Figure 10:**
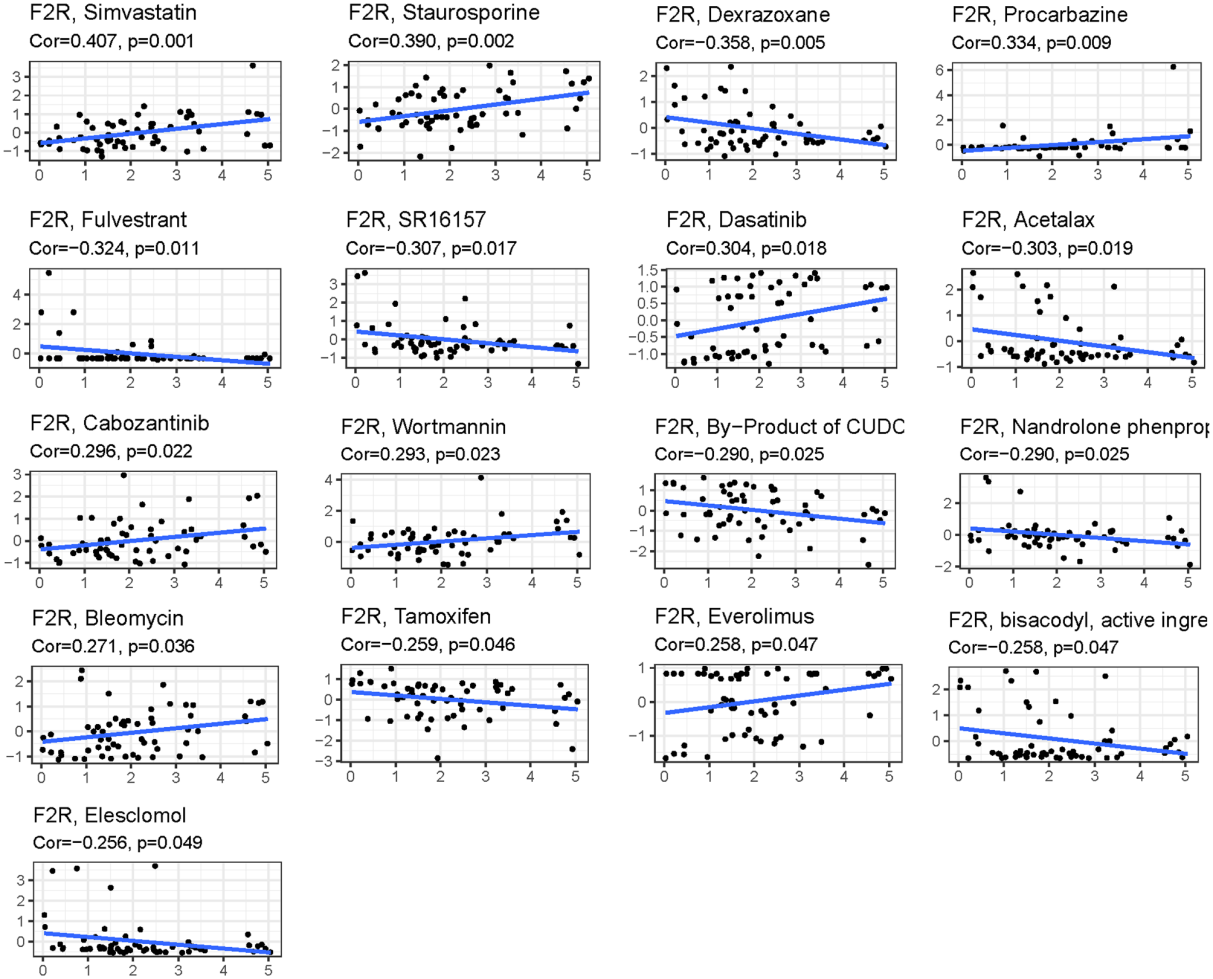
Correlation plot of *F2R* gene and drug sensitivity. (**A**) *F2R* expression was positively correlated with drug sensitivity. (**B**) *F2R* expression was negatively correlated with drug sensitivity. The x-axis is gene expression and the y-axis is drug sensitivity.

The sensitivity of the *F2R* gene to commonly used antitumour drugs was investigated using the CellMiner database. The relationship between gene expression and drug sensitivity was calculated, revealing a significant association of *F2R* expression with the sensitivity of 14 drugs (Fig. 10). Among them, *F2R* was positively associated with Simvastatin, Staurosporine and Procarbazine and negatively associated with Dexrazoxane, Fulvestrant and SR16157. Notably, some of these drugs, exhibiting sensitivity to *F2R* expression level, have yet to undergo extensive medical testing. Their potential as promising candidates for future therapeutic interventions warrants further exploration.

### The potential of predicting immunotherapy efficacy based on *F2R* levels in tumors

**Figure 11:**
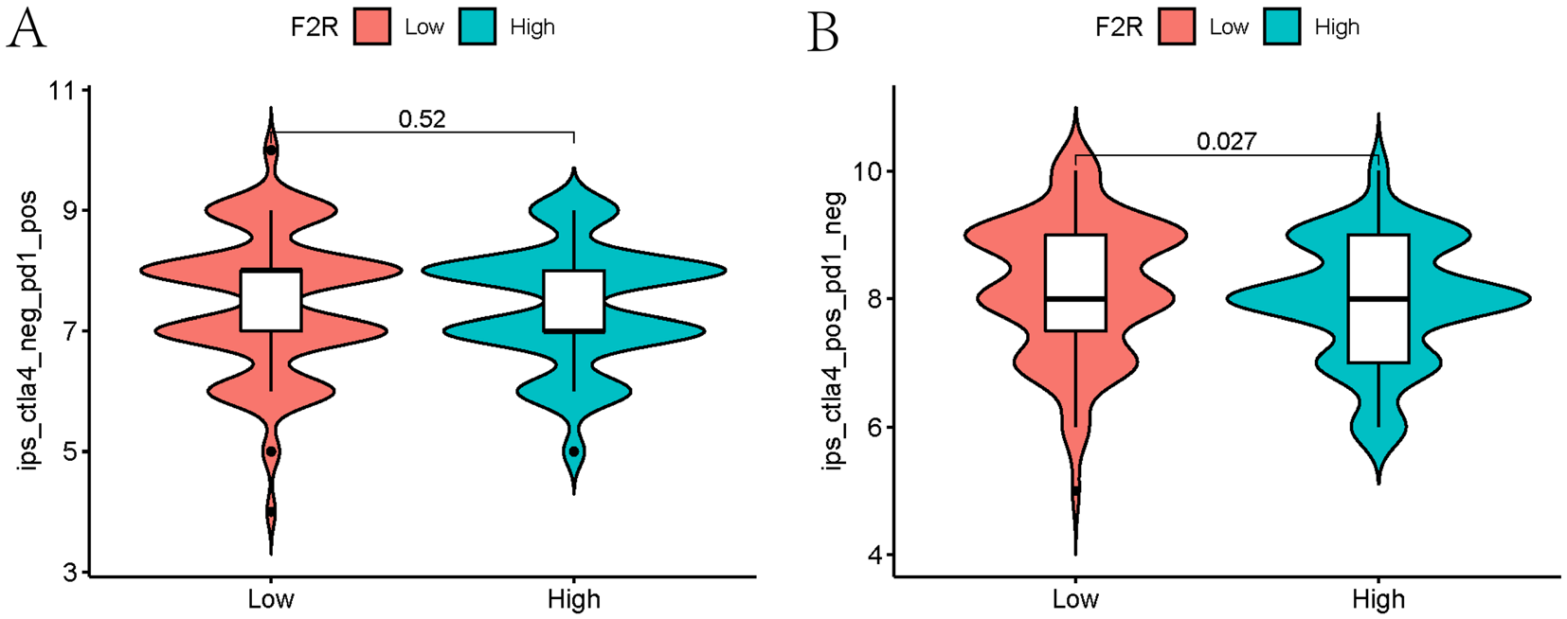
Immunoscore of *F2R* expression with anti-pd1 and anti-ctla4 treatment. (**A**) High and low immune scores in the low and high *F2R* expression groups while on anti-pd1 therapy. (**B**) High and low immune scores in the low and high *F2R* expression groups while on anti-ctla4 therapy.

To investigate the potential of utilizing *F2R* level as a predictive biomarker for immunotherapy efficacy in STAD, we conducted a comprehensive investigation leveraging established immunotherapy biomarkers’ predictive capabilities. Our investigation focused on evaluating *F2R*’s role in shaping the immunotherapeutic response in STAD. We examined the relationship between high and low expressions of *F2R* and immune scores during anti-PD1 and anti-CTLA4 treatment. Our results indicated no significant difference between the low and high *F2R* expression groups receiving anti-PD1 treatment. However, when subjected to anti-CTLA4 treatment, patients in the low *F2R* expression group exhibited higher immune scores, suggesting a more favorable outcome for individuals with low *F2R* expression when undergoing anti-CTLA4 treatment (Fig. 11A,B).

### Impact of *F2R* on the proliferation, migration and invasion of STAD cells

**Figure 12:**
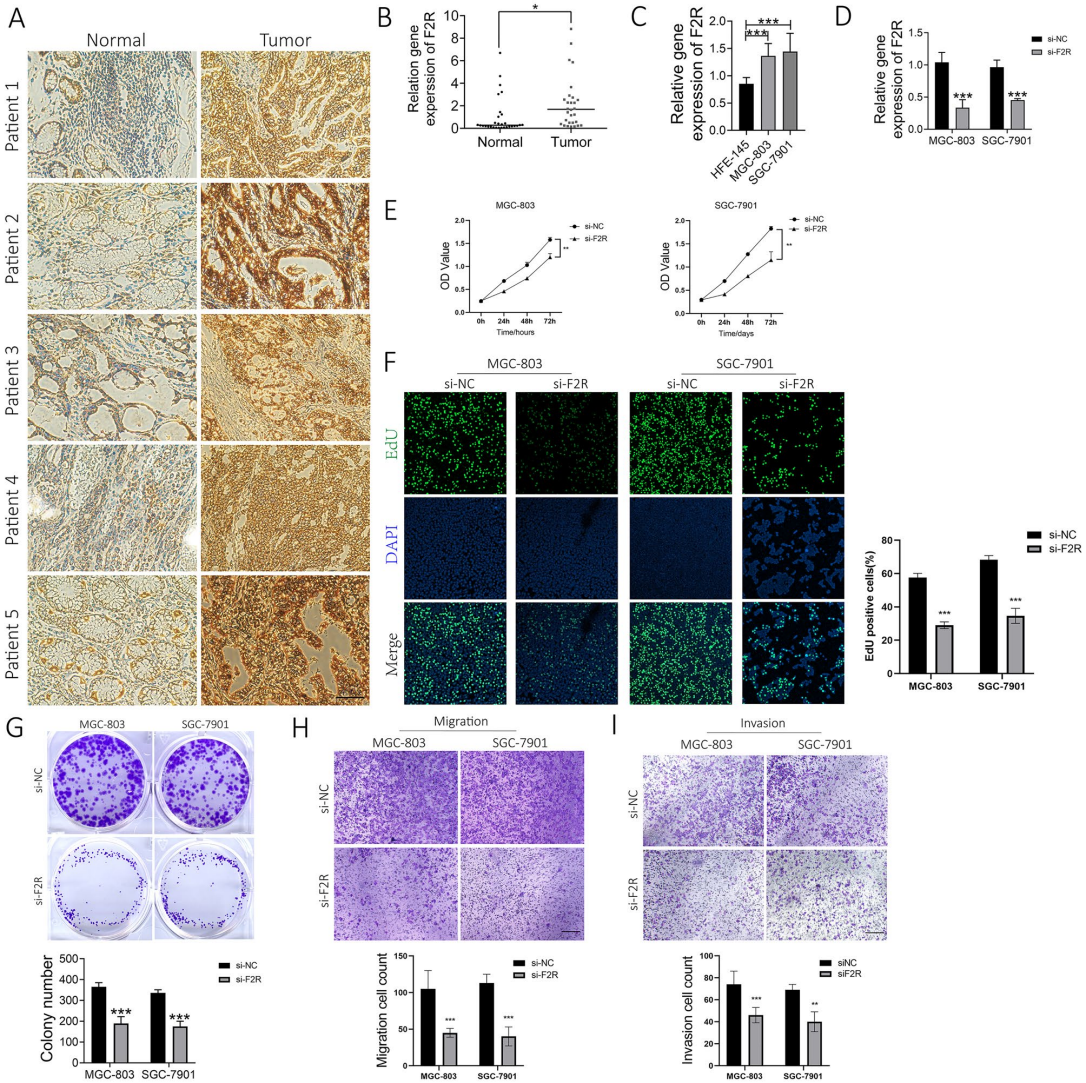
*F2R* promotes cell proliferation, migration and invasion in GC cells. (**A**) IHC staining analysis of *F2R* protein expression in matched gastric adenocarcinoma tissues (T) and adjacent non-cancerous tissues (N) (× 400 magnification). (**B**) Levels of *F2R* mRNA in 30 pairs of carcinomatous and paracarcinomatous tissues from GC patients detected by qRT-PCR. (**C**) Relative *F2R* mRNA expression in normal gastric cells (HFE-145) and gastric cancer cell lines (MGC-803 and SGC-7901) determined by RT-qPCR. (**D**) RT-qPCR was used to detect the inhibitory efficiency of *F2R* siRNA in MGC-803 and SGC-7901 cells. (**E**) CCK-8 assay was used to detect the viability of cells after *F2R* siRNA transfection. (**F**) The effects of *F2R* siRNA on the proliferation of MGC-803 and SGC-7901 cells were detected using EdU assays. (**G**) The effects of *F2R* siRNA on the proliferation of MGC-803 and SGC-7901 cells were detected using colony formation assays. (**H,I**) Transwell assay was used to determine the effects of *F2R* siRNA on MGC-803 and SGC-7901 cell migration and invasion. *p < 0.05, **p < 0.01, ***p < 0.001 vs. si-NC.

To further validate *F2R* expression in gastric adenocarcinoma, we performed IHC staining on human GC tissue. This analysis revealed significantly upregulated *F2R* expression in five paired samples compared to their matched non-cancerous counterparts (Fig. 12A). qRT-PCR confirmed that *F2R* was significantly elevated in gastric cancer tissues, which was consistent with the IHC results (Fig. 12B). The intense staining observed in tumor tissues provides compelling evidence for *F2R*’s heightened role in gastric cancer, paving the way for further investigation into its potential clinical implications. To verify the expression level of *F2R* in STAD cell lines, we assessed *F2R* expression in STAD cell lines (MGC-803 and SGC-7901) and normal gastric cells (HFE-145) using qPCR. *F2R* exhibited a significant upregulation (p < 0.05) in MGC-803 and SGC-7901 compared to the normal gastric cell line HFE-145 (Fig. 12C). Following *F2R* siRNA transfection, the downregulation of *F2R* in MGC-803 and SGC-7901 cell lines was confirmed through qPCR (Fig. 12D). We further investigated the impact of *F2R* on tumor progression. CCK-8 assays revealed a significant reduction in cell viability upon *F2R* silencing (Fig. 12E), suggesting its role in promoting cell survival. This anti-proliferative effect was further corroborated by EdU and colony formation assays, showcasing decreased proliferation and colony-forming ability in *F2R*-depleted cells (Fig. 12F,G). To investigate the effects of *F2R* on the migration and invasion capabilities of MGC-803 and SGC-7901 cells, we performed a transwell assay with cells transfected with *F2R* siRNA. As shown in Fig. 12H,I *F2R* knockdown significantly inhibited the migration and invasion of MGC-803 and SGC-7901 cells compared with the negative control group. In conclusion, our in vitro experiments demonstrated that *F2R* promoted the proliferation, migration and invasion of MGC-803 and SGC-7901 cells. This supports its potential as a novel therapeutic target specifically for STAD, offering opportunities for inhibiting tumor progression.

## Discussion

Exploring biomarkers and molecular targets is imperative for early diagnosis and effective treatment of STAD^38^, a prevalent malignancy within the digestive system. Through robust bioinformatics analyses of the TCGA database, we identified genes associated with STAD pathogenesis and clinical prognosis. Our investigation revealed a significant upregulation of *F2R* in STAD, prompting compelling inquiries about its specific role in the development and progression of this cancer subtype. Compared to other gastrointestinal tumors, the elevated expression of *F2R* in STAD suggests its unique function within STAD biology. This finding has ignited a quest to decipher whether *F2R*’s role in STAD is distinct from its functions in other cancers. This distinction is pivotal for unraveling the complexities of STAD and holds the potential to unveil novel therapeutic avenues tailored to its specific characteristics.

The prognostic significance of *F2R* expression in STAD provides new perspectives for patient stratification and treatment approaches. Our investigation revealed a strong association between high *F2R* expression and poorer survival outcomes, high *F2R* expression is associated with poor prognosis of GC, *F2R* gene is an oncogene, when it is highly expressed in tissues, it promotes the malignant progression of GC cells and shortens the survival time of patients, positioning *F2R* as a promising prognostic biomarker. Recognizing this correlation underscores the importance of considering *F2R* expression in therapeutic decision-making and emphasizes its potential as a guiding factor in the development of targeted therapies. In a thorough examination of *F2R*’s impact on clinicopathological parameters in STAD patients, our findings indicated that *F2R* serves as an independent prognostic factor influencing survival in GC patients. This aligns with existing literature, where *F2R* has been consistently linked to increased tumor aggressiveness and unfavorable prognosis across various cancer types. Incorporating *F2R* expression levels into established prognostic models has the potential to enhance the precision of survival prediction, offering valuable insights for personalized treatment planning in STAD patients. The high expression of *F2R* in STAD not only signifies its potential as a prognostic marker but also hints at its involvement in tumorigenesis and early progression. The correlation between *F2R* level and advanced disease staging in STAD suggests a role for *F2R* in driving tumor progression and metastasis. Investigating the role of *F2R* in tumor development and its potential as a biomarker for early detection could provide insights into the temporal changes in *F2R* expression from early to late stages of STAD.

The correlation of *F2R* with key cancer-related signaling pathways suggests that *F2R* inhibition could disrupt these pathways, thereby inhibiting tumor proliferation and invasion. To explore this avenue further, the development of inhibitors or monoclonal antibodies against *F2R* could offer a novel therapeutic approach for STAD. The potential synergies between *F2R*-targeted therapies and existing treatment modalities such as chemotherapy and immunotherapy are worth exploring. This may lead to more effective combination therapies that enhance therapeutic efficacy while minimizing side effects. Furthermore, delving into the role of *F2R* within the tumor microenvironment (TME), including its interactions with immune cells and immune checkpoints, unveils opportunities for immunomodulatory strategies. Targeting *F2R* could potentially reshape the dynamics between tumor and immune system, thereby improving the efficacy of immunotherapy for STAD. As research progresses, the translation of these findings into clinical applications becomes critical. Investigating clinical treatment strategies for *F2R*-targeted therapies will provide essential evidence regarding efficacy and safety. This could pave the way for new treatment options for STAD patients.

The impact of immune infiltration on tumor behavior and prognosis underscores the potential of targeting the TME as a promising avenue for cancer treatment. In this study, we focused on understanding the role of *F2R* in immune infiltration, revealing a significant correlation between increased *F2R* expression and decreased TMB. As an immune-related gene, *F2R* is known to influence the behavior of immune cells, prompting a comprehensive analysis of its effects on the TME using the CIBERSORT algorithm. The CIBERSORT results demonstrated that high *F2R* expression in STAD is associated with the activation of memory CD4^+^ T cells, quiescent dendritic cells, eosinophils, M2 macrophages and M1 macrophages. Conversely, low *F2R* expression correlated with activated NK cells. The activation of CD4^+^ T cells, known for their role in regulating cytolytic mechanisms and enhancing B cell and CD8^+^ T cell responses^39,40^, suggests a complex interplay in STAD progression. The activation of macrophages and their subtypes in the high *F2R* expression group aligns with previous studies, implicating these cells in cancer development and metastasis^41^.

Inflammation can be divided into two categories-acute inflammation and chronic inflammation—depending on the duration of the disease. Acute inflammation is the initial response to a noxious stimulus and lasts for days or weeks. Most of the infiltrated inflammatory cells are granulocytes. If the pro-inflammatory stimulus is not eliminated during the acute inflammation, the result will be chronic inflammation. The main infiltrating immune cells at the site of chronic inflammation are macrophages and lymphocytes. Large numbers of immunosuppressive cells inhibit the killing function of T cells, leading to immune escape, which promotes tumour formation. There is evidence that up to 25% of cancers are associated with chronic inflammatory diseases, some of which are clinically considered precancerous, and our study suggests a potential link between *F2R* expression and the promotion of chronic inflammation in STAD. The persistence of inflammatory factors and immune cells, particularly macrophages, in the microenvironment may contribute to immune escape, fostering tumor formation and progression^42^. This suggests that increased *F2R* expression promotes the development of chronic inflammation, which in turn promotes increased immune pathway activity. Therefore, activation of immune pathway activity in GC patients is associated with poor prognosis in patients.

The association between *F2R* and immune pathways suggests its potential role as a key regulator in the immune response. In both in vivo and in vitro experiments, *F2R* was found to be significantly upregulated in gastric adenocarcinoma tissues. The high expression of the *F2R* gene promotes the progression of cancer, and the progression of cancer will activate tumor-related immune cells to produce inflammatory responses, resulting in poor prognosis in patients with high expression of the *F2R* gene^43^. Therefore, we speculate that *F2R* is a poor prognostic factor for patients with STAD and one of the key factors for regulating the immune response. In this study, *F2R* was proven to be related to the infiltration of immune cells in the STAD microenvironment. Moreover, knockdown of *F2R* resulted in a significant inhibition of cell proliferation, migration, and invasion in MGC-803 and SGC-7901 cells. Collectively, these bioinformatics analyses and experimental results position *F2R* as a potential biomarker and therapeutic target in STAD.

While our study enhances our understanding of the role of *F2R* in GC, certain limitations should be acknowledged. Firstly, the limited sample size and the absence of specific details on surgery, chemotherapy, and tumor size limit the completeness of our analysis. Secondly, reliance on public databases and published articles introduces potential biases, and data quality could influence results. Thirdly, the precision of the database and the choice of statistical methods can impact result interpretation. However, the consistency of our results across multiple databases and experimental validations reinforces the robustness of our conclusions. In the field of GC biomarker screening, global studies often encounter issues such as small sample sizes, reliance on single data analysis methods, and a lack of experimental verification. To address these challenges, our study employs a comprehensive approach, integrating various bioinformatics methods for an in-depth analysis of gene datasets from multiple platforms. By expanding the sample size and corroborating findings with in vitro cell experiments, we aim to enhance the scientific rigor of bioinformatics analysis. This endeavor provides a more accurate exploration of GC pathogenesis and therapeutic targets, offering a molecular biology foundation and suggesting new avenues for subsequent experimental studies.

## Conclusion

We used bioinformatics to explore the relationship between high *F2R* gene expression and GC progression. We also evaluated the feasibility of *F2R* gene expression as a prognostic factor for GC and predicted potential signaling pathways through which *F2R* affects GC progression. Our findings provide novel insights into the prevention and prognosis of GC. The construction of a nomogram comprehensively visualized all factors in the model, providing predictions that closely aligned with real-world scenarios. Calibration curves, assessing the probability of survival at 1, 3, and 5 years, confirmed the accuracy of the nomogram, showcasing strong agreement between observed and predicted OS in GC patients. These findings suggest that high *F2R* expression may independently contribute to a heightened risk of poor prognosis in GC. *F2R*’s interaction with *hsa-miR-144-5P* and *lncFTX*, as well as with *hsa-miR-486-5P* and *lncZEB1-AS1*, forms a comprehensive lncRNA-miRNA-mRNA ceRNA network, implicating *F2R* in GC development. The analysis of this network provides initial insights into *F2R*’s role in the onset and progression of GC. GSEA identified the signaling pathway associated with *F2R* mRNA expression in GC, revealing a significant association with tumor progression. The results suggest that suppressing *F2R* expression could potentially inhibit the progression of GC cells. The prediction of *F2R*’s impact on GC through GSEA indicates its involvement in activating the MAPK and JAK-STAT pathways linked to tumor progression. Future therapeutic strategies may involve inhibiting these pathways to impede gastric cancer progression. This study underscores *F2R*’s potential as a prognostic biomarker for GC, positioning it as a promising predictive biomarker and a potential target for immunotherapy. Subsequent experimental investigations are crucial to validate the biological mechanisms underlying the impact of elevated *F2R* expression on gastric cancer progression.

## Methods

### Gene expression level analysis

GEPIA2 (Gene Expression Profiling Interactive Analysis) is a web tool based on TCGA and GTEx data that provides fast and customizable functionality. The GEPIA database was used to search and analyze *F2R* gene expression in GC and adjacent tissues, as well as OS data from different cancer datasets. The screening criteria |Log^Change^| > 1 and p < 0.01 were set as significance thresholds. Cancer type is selected as STAD, and we used log_2_^(TPM+1)^ for logarithmic scaling. The Jitter Size was 0.4. Within the GEPIA online database, the STAD dataset was chosen for further investigation. We retrieved a survival graph illustrating the correlation between *F2R* expression and OS in the form of Kaplan–Meier curves. The median filter was applied with a truncation value, and hazard ratio was calculated based on the Cox Proportional-Hazards (PH) model, with 95% confidence intervals (CI) depicted as dotted lines. Axis units were specified in months. For PPI analysis, the top 20 genes displaying the most significant correlation with *F2R* were selected from the STAD dataset within the GEPIA database. Timer 2.0 (http://timer.cistrome.org/) analysis was used to explore the association between tumor immune cell infiltration and *F2R* expression levels. This included B cells, CD8^+^ T cells, CD4^+^ T cells, neutrophils, macrophages and dendritic cells. The TIMER database, a web server built on the R Shiny Web framework, was used for the exploration module. TCGA tumor data from TIMER allowed us to study the expression of *F2R* in the TCGA-STAD cohort, visualizing it through the visualization function of the website. In the Gene_DE module, the differential expression of *F2R* between tumor and adjacent normal tissues was studied across all TCGA tumors. The distribution of gene expression levels is shown using box plots. The statistical significance of Wilcoxon test calculations is annotated with stars (*p values < 0.05; **p value < 0.01; ***p value < 0.001). The gene module in TIMER focused on *F2R*, visualizing its correlation with immune cell infiltration in the STAD dataset. By clicking on different immune cells on the heatmap, the scatter plot illustrated the relationship between estimated immune cell infiltration and *F2R* gene expression. Partial Spearman correlations were used for association analysis, taking into account the confounding factor of tumor purity, with the purity adjustment option selected.

### Data mining of the TCGA database

The analysis included RNA-Seq HTSeq count gene expression data and clinical information obtained from GC patients, using R software (version 4.2.1). The TCGA database was the source for GC samples, containing clinical information, mRNA, lncRNA and miRNA expression data. All original RNA sequence data, including lncRNA, miRNA, and mRNA, were obtained from the TCGA database. mRNA information from 375 GC tissues and 32 adjacent nontumor tissues was obtained from the TCGA database. High-quality mRNA information was extracted, followed by name conversion and logarithmic correction of RNA-seq data obtained from both TCGA and GEO. The R software packages ggplot2, limma and beeswarm were used to statistically analyze the expression of *F2R* in the two groups. Scatter plots and pairing plots were generated to visually display the results. Clinical data on GC patients, including age, gender, clinical grade, T stage, M stage, and N stage, were also acquired from the database. The Perl programming language was used to match gene expression information with clinical information. Unknown or incomplete clinical information was removed from the dataset. The integrated dataset, combining patient clinical information with *F2R* expression data, underwent further statistical analysis using the ggpubr package in R software. The survival package in R was used to analyze the survival status and gene expression. The significance level for all analyses was set at p < 0.05.

### Logistic regression analysis

Clinical data of STAD patients were downloaded from the TCGA database, and the patients’ clinical information was combined with *F2R* expression data. Logistic regression analysis was performed using R software to analyze the patients’ clinical data. p < 0.05 was considered to be statistically significant.

### Independent prognostic analysis

In our investigation into the impact of *F2R* expression on the prognosis of STAD patients, we employed a comprehensive approach. Univariate Cox regression analysis was initially applied to quantify the correlation between *F2R* expression levels and patient survival. Following this, multivariate analysis was conducted to determine whether *F2R* stands as an independent prognostic factor for survival in STAD patients. The patients’ Cox data were statistically analyzed using the survival package within the R software, employing both univariate and multivariate methods to ascertain independent prognostic indicators. The results were visualized using forest plots. Clinical data of STAD patients were downloaded from the TCGA database, and the patients’ survival time and status were integrated with *F2R* expression data. The R software survival, survival, and time ROC packages were then used to conduct a rigorous time-dependent receiver operating characteristic (ROC) analysis. This analysis served to validate the predictive efficacy of the risk model at distinctive time points: 1, 3, and 5 years. The significance threshold was set at p < 0.05.

### GSEA

The GSEA software, downloaded from the GSEA website, is a powerful platform for gene set enrichment analysis. The initial molecular signature database comprises 1325 gene sets, spanning various categories such as biological pathways, chromosomal locations, upstream cis-patterns, drug therapy responses, and expression profiles from microarray datasets. Additional gene sets can also be created through genetic and chemical perturbations, computational analysis of genomic information and other biological annotation. GSEA results yield a computational enrichment score (ES), indicating the extent to which a gene set S is overrepresented in the extreme (top or bottom) of the overall sorted list L. To enhance result interpretation, the ES is normalized to account for genome size, resulting in a normalized enrichment score (NES). To control for false positives, the false positive rate (FDR) corresponding to each NES is calculated. Patient data were stratified into high and low *F2R* gene expression groups based on the median level. These TCGA data were formatted in text files and imported into GSEA software. The grouping and expression matrix files were then imported into GSEA 4.3.2 lineage enrichment analysis. The resulting data were exported to analyze significant functional and pathway differences between the high and low *F2R* expression groups. The genomic alignment was repeated 1000 times for each analysis. p values, FDR values, ES values, and NES values of GSEA were analyzed. In the gene enrichment analysis, expression differences were considered significant if they met the criteria of p < 0.05 and FDR < 25%.

### UCSC Xena

The UCSC Cancer Genomics Browser (https://genome-cancer.ucsc.edu/) is a web-based application that integrates relevant data, analysis, and visualization to facilitate the exploration and sharing of research observations. The platform can be used to investigate the relationship between genomic alterations and phenotypes by visualizing a variety of omics data, and phenotypic characteristics such as age, subtype classification, and genomic biomarkers. The Cancer Genomics Explorer currently contains 575 public datasets derived from genome-wide analyses of more than 227,000 samples. These datasets include information from TCGA, CCLE, Connectivity Map, and TARGET. For this study, data on the relationship between *F2R* gene expression levels and their copy number variations were downloaded from the Genome Data Public Center within the UCSC Xena browser. A total of 415 STAD and 35 matched normal samples were extracted for further analysis. To better understand the functions of *F2R*, its expression data across multiple cancer types (pancancer) were also extracted. The ggpubr package in R software was used to visualize the expression levels of *F2R* in tumor and noncancerous tissues across various cancers. The statistical significance of the analyses was determined at a significance level of p < 0.05.

### Construction of the ceRNA network

The miRNA gene expression data from normal gastric tissue and GC tissue samples were downloaded from the TCGA website. Then, the mirbase (https://www.mirbase.org/) database was utilized to obtain miRNA gene IDs, facilitating ID conversion for extracting miRNA expression levels in GC tissues and noncancerous tissues. StarBase v2.0 (https://starbase.sysu.edu.cn/index.phpStarBase) was used to build a comprehensive network of microRNA–lncRNA interactions. Utilizing a dataset of 108 CLIP-Seq generated from 37 independent studies, starBase v2.0 systematically identifies RNA-RNA and protein-RNA interaction networks. This database, enriched with CLIP-Seq experimental support, facilitated the identification of more than 10,000 ceRNA pairs from miRNA target sites. To predict the functions of miRNAs and other non-coding RNAs (ncRNAs), 13 functional genome annotations were incorporated, and miRFunction and ceRNAFunction web servers were developed. An interactive web implementation was also developed to facilitate visualization, analysis, and download of these large-scale datasets. Information on miRNAs and lncRNAs related to *F2R* was screened based on stringent criteria such as mammalian data, human genome version hg19, minimum CLIP data rigor (≥ 5), and presence or absence of degradome data. The STARBase database predicted interactions among lncRNA/miRNA pairs and miRNA/mRNA pairs. Cytoscape software (version 3.6.1) was employed to construct a lncRNA–miRNA–miRNA–ceRNA mRNA regulation network, unraveling intricate lncRNA–miRNA and miRNA–mRNA relationships. To construct miRNAs network regulated by *F2R*, miRNAs targeted by *F2R* were identified using starBase v2.0. Leveraging the regulatory mechanisms of ceRNA networks, miRNAs expression differences between normal and tumor samples were analyzed. Spearman statistical analysis identified miRNAs negatively correlated with *F2R* mRNA expression. The STARBASE database was again utilized to screen upstream miRNAs of mRNA, and the selected miRNAs were analyzed for correlation, expression differences, and survival in R software. Spearman statistical analysis showed that there was a correlation between miRNA and mRNA. miRNAs were screened according to cor <  − 0.2 and p < 0.001 screening criteria. lncRNA regulating miRNA and *F2R* was screened by STARBASE v2.0. According to the regulatory mechanism of the ceRNA network, lncRNA expression was different in normal samples and tumor samples, and lncRNAs were highly expressed in tumor tissues. Spearman statistically analysed whether lncRNAs were correlated with miRNAs and mRNAs. According to the screening criteria of COR = 0.2 and p = 0.001, lncRNAs were negatively correlated with miRNAs, and lncRNAs were positively correlated with mRNAs. This analytic process, involving multiple databases, computational tools, and statistical analyses, and the selected lncRNAs met the requirements of negative correlation with miRNA expression, positive correlation with mRNA expression, high expression in tumor tissues, and negative correlation with patient survival time. The above differences were statistically significant. The resulting lncRNA, miRNA and mRNA networks were imported into Cytoscape 3.8 to map the ceRNA networks. Led to the identification of key regulatory networks involving *F2R*, miRNAs, and lncRNAs. The resulting networks were visualized using Cytoscape 3.8, providing a comprehensive representation of the ceRNA interactions and shedding light on the intricate regulatory mechanisms surrounding *F2R* expression in GC.

### Immunocorrelation analysis

The R software limma package and the ggpubr package were used to investigate the relationship between *F2R* expression and tumor-infiltrating immune cells. We leveraged the Tumor Immune Estimation Resource (TIMER2.0), a web-based interactive platform, to systematically analyze the immune infiltration in various malignant tumors. The TIMER2.0 database uses six advanced algorithms to deliver a rigorous assessment of tumor-infiltrating lymphocyte (TIL) levels. This platform is particularly valuable for analyzing TCGA or tumor-related data and provides accurate estimates of tumor purity. Our investigation into the expression of *F2R* in GC involved an in-depth examination of its relationship with TILs using gene modules. In addition, we also analyzed the relationship between *F2R* expression and TIL gene markers through correlation modules. These gene markers encompassed various immune cell types, including CD8^+^/CD4^+^ T cells, B cells, monocytes, NK cells, DCs, tumor-associated macrophages (TAMs), M1 macrophages, M2 macrophages, and neutrophils. Utilizing the correlation module, we generated a discrete map illustrating the expression patterns between a pair of custom genes specific to GC. The correlation and Spearman estimates were analyzed for statistical significance. The expression levels of the genes were reported in log_2_^RSEM^ (RSEM: RNA-Seq by Expectation–Maximization).

### Immune microenvironment landscape and drug sensitivity analysis

Estimates of stromal and immune cells infiltration were determined using the estimation algorithm, and the CIBERSORT algorithm was used to estimate the scores of 22 distinct immune cells types. Correlation analysis between *F2R* expression and cancer type, as well as immune/stromal score and immune cell type, was performed using the ggplot2 package, with statistical significance set at p < 0.0001. Correlations between *F2R* expression and TMB were analyzed and visualized using the limma package, ggplot2 package, ggpubr package, and ggExtra package in R software. Drug sensitivity, a critical metric represented by the IC_50_ value, is an essential indicator for evaluating the efficacy of drug response. Based on the Genomics of Drug Sensitivity in Cancer (GDSC) database, the antitumour drug response of each GC sample was predicted by the R package pRRophetic, stratifying the analysis into low and high-risk categories. All statistical analyses were visualized by the ggplot2 R package.

### GO and KEGG enrichment analyses

To elucidate the underlying biological processes and pathways associated with *F2R*, we used stringent criteria (|fold change| ≥ 2, p < 0.05) to identify genes correlated with *F2R* expression. Then, the R software packages, including clusterProfiler, org.hs.eg.db, enrich lot, ggplot2, circle, RColorBrewer, dplyr, and ComplexHeatmap, were used to conduct a comprehensive enrichment analysis and visually represent GO and KEGG^44–46^.

### PPI network construction

PPI networks were extracted from the STRING database. The STRING database facilitates the collection of both direct (physical) and indirect (functional) interactions among proteins, providing a nuanced understanding of their relationships. To gain insights into the *F2R*-regulated protein interacting network, we constructed the *F2R* PPI network with a minimum interaction score of 0.1. The predictions regarding *F2R*-interacting proteins were predominantly derived from similar genes identified in the GEPIA database. By cross-referencing these databases, we aimed to bolster the robustness of our findings and provide a more comprehensive overview of the proteins potentially influenced by *F2R*.

### DNA methylation analysis of CpG islands within the *F2R* gene

We explored the methylation status of the *F2R* gene using the MethSurv database (http://biit.cs.ut.ee/methsurv/). This web tool utilizes TCGA data to enable comprehensive survival analysis. Within the TCGA-STAD dataset, we specifically focused on the CpG loci of the *F2R* gene to analyze their methylation patterns. The association between the prognostic potential and OS of the CpG methylation status of *F2R* was analysed in TCGA-STAD samples.

### Clinical sample collection

GC tissue samples and adjacent non-tumour tissues from 30 pairs of patients were collected at the Department of Gastrointestinal Surgery, Second Affiliated Hospital of Wannan Medical College (Wuhu, Anhui, China). The diagnosis of GC in the surgical specimens was independently determined by two senior pathologists. None of the patients had received any treatment prior to surgery. The study was approved by the Ethics Committee of the Second Affiliated Hospital of Wannan Medical College, and informed consent was obtained from all patients. The study was conducted according to the Declaration of Helsinki.

### Immunohistochemical (IHC) staining

GC tissue samples and adjacent non-tumour tissues were collected from 30 pairs of patients who underwent GC surgery at the Department of Gastrointestinal Surgery. Five pairs of tissues were randomly selected for immunohistochemical staining. The tissues were first fixed in 4% paraformaldehyde. They were then dehydrated. Sections of 4 mm thickness were then cut using a rotary slicer (RM2255, Leica, Germany). Immunohistochemistry for *F2R* protein was performed. Tissues were deparaffinised by immersion in xylene and rehydrated in reduced concentrations of ethanol after baking at 60 °C for 2 h. The sections were incubated with *F2R* primary antibody (1:100, ER1917-72, Huaan Biotechnology Co., Limited, China) for 12 h at 4 °C. The next day, the sections were rinsed with PBS and incubated with biotinylated secondary antibody for 2 h at room temperature. Finally, slides were treated with ImmunoPure Metal Enhanced DAB Substrate Kit (Pierce, Rockford IL) according to the manufacturer’s instructions and images were captured using a Nikon E800 microscope.

### Cell culture and plasmid transfection

The GC cells MGC-803 and SGC-7901 were used in this study. These cells were maintained in RPMI 1640 medium supplemented with 10% fetal bovine serum (FBS) under a humidified atmosphere of 5% carbon dioxide at 37 °C. To investigate the functional role of *F2R*, we employed RNA interference technology. Specifically, cells were transfected with either negative control siRNA (si-NC) or small-interfering RNAs against *F2R* (si-*F2R*). The siRNA sequence was GAUUAGUCUCCAUCAAUAATT. All siRNAs were purchased from GenePharma (Shanghai, China).

### Quantitative PCR

Total RNA was extracted using TRIzol reagent (Invitrogen, USA), following the manufacturer’s instructions. Complementary DNA (cDNA) was synthesized using a commercially available kit (Ambion; Thermo Fisher Scientific, Inc). Subsequently, qPCR was performed using a commercial kit, and each experiment was repeated 3 times. The primers for qPCR were as follows: *F2R* (forward primer: 5ʹ-CCACCTTAGATCCCCGGTCAT-3ʹ, reverse primer: 5ʹ-GTGGGAGGCTGACTACAAACA-3ʹ) and GAPDH (forward primer: 5ʹ-AAAGCCTGCCGGTGACTAA-3ʹ, reverse primer: 5ʹ-AGAGTTAAAAGCAGCCCTGG-3ʹ). *GAPDH* was used as a reference gene for normalization of *F2R* mRNA expression.

### Cell proliferation assay

Logarithmically growing cells were seeded into a 96-well plate and treated with a 50 µM 5-ethynyl-2ʹ-deoxyuridine (EdU) stock solution at 37 °C for 2 h. The cells were then fixed with 4% paraformaldehyde (PFA), and the nuclei were stained with DAPI (D9542, Sigma-Aldrich). The resulting fluorescence was captured using an Olympus microscope. Cell proliferation was assessed with a CCK-8 assay kit. Briefly, cells were cultured in a 12-well plate for 3 days before the cell proliferation assay. Then 100 ml of CCK-8 solution was added to the 12-well plate, after which an appropriate volume of stop solution was added to stop the reaction. The plate was read using a microplate analyzer to measure the optical density (OD) of the supernatant after a 2-h incubation period. MGC-803 and SGC-7901 cells, transfected either with negative control siRNA or *F2R* siRNA, were seeded in 6-well plates at a density of 4000 cells per well. After approximately 2 weeks, the medium was discarded, and the colonies were fixed with 4% paraformaldehyde for 30 min. Subsequently, crystal violet staining was performed for 15 min at room temperature, followed by rinsing with PBS.

### Cell migration and invasion assays

Cell migration assays were performed using 24-well cell culture plates with 8 μm non-porous membranes (Millipore, USA). Cells (5 × 10^4^) were resuspended in 200 μl serum-free medium in the upper chamber. The lower chamber was supplemented with 600 μl DMEM medium containing 20% FBS. Following 24 h of incubation at 37 °C and 5% CO_2_, the cells on the upper surface were gently scraped. The cells that had migrated to the lower surface were fixed with 4% paraformaldehyde for 30 min and then stained with a crystal violet solution for 20 min at room temperature. For the Matrigel invasion assay, the upper chamber of CIM plate 16 was pre-coated with diluted Matrigel (356234, BD Biosciences). The same procedures were then followed.

### Statistical analysis

Statistical analysis was performed using GraphPad Prism 9.0 and R (version 4.2.1). The analysis involved the utilization of the limma package, ggplot2 package, and ggpubr package. Paired and unpaired samples were assessed for the expression of *F2R* using the Wilcoxon signed rank test and Wilcoxon rank sum test, respectively. The relationship between *F2R* expression and clinical features was analyzed using the limma packages and ggpubr packages. Univariate and multivariate analyses of Cox proportional hazard regression models were used to identify independent prognostic factors, including gender, age, stage T, N, M, pathological stage and *F2R* expression, in assessing the risk of death. The CIBERSORT algorithm was applied to predict the abundance of 22 infiltrating immune cells in the TME using the GC gene expression profile downloaded from the TCGA database. At the same time, the ESTIMATE algorithm was used to obtain the matrix/immunity scores of the above datasets. The results were visualized using the limma package, ggplot2 package, and ggpubr package. The Wilcox test was used to compare stromal/immune scores among different clinicopathological groups, with a significance threshold set at p < 0.05.

### Supplementary Information

Supplementary Information.

Supplementary Table S1.

Supplementary Table S2.

Supplementary Table S3.

Supplementary Table S4.

Supplementary Table S5.

Supplementary Table S6.

Supplementary Table S7.

Supplementary Table S8.

## Data Availability

All data generated or analysed for this study are included in this article. Further details are available from the corresponding author upon request. The datasets generated and/or analysed during the current study are available in the The Cancer Genome Atlas (TCGA-STAD) datasets (https://portal.gdc.cancer.gov/repository), Gepia datasets (http://gepia.cancer-pku.cn/repository), TIMER2 datasets (http://timer.cistrome.org/repository), GEO datasets (https://www.ncbi.nlm.nih.gov/geo/repository), HPA datasets (https://www.proteinatlas.org/repository), MethSurv database (http://biit.cs.ut.ee/methsurv/repository), mirbase datasets(https://www.mirbase.org/repository), UCSC Xena datasets (http://xena.ucsc.edu/repository) and the CellMiner datasets (https://discover.nci.nih.gov/cellminer/home.do/repository).

## Abbreviations

*F2R*: Coagulation factor II (thrombin) receptor
TCGA: The Cancer Genome Atlas database
STAD: Stomach adenocarcinoma
GSEA: Gene set enrichment analysis
HR: Hazard ratio
TNM: Tumour staging
lnc RNA: Long noncoding RNA
miRNAs: MicroRNAs
GO: Gene Ontology
KEGG: Kyoto Encyclopedia of Genes and Genomes
ROC: Receiver operating characteristic
PPI: Protein interaction
