# Supplementary material for: Bioinformatics analysis identifies coagulation factor II receptor as a potential biomarker in stomach adenocarcinoma
Source: Sci Rep. 2024 Jan 30;14:2468. doi: 10.1038/s41598-024-52397-6 (PMC10827804; doi:10.1038/s41598-024-52397-6)
Supplement: Supplementary file 1 — Supplementary Information. [file 41598_2024_52397_MOESM1_ESM.docx]

**Bioinformatics analysis identifies coagulation factor II receptor as a potential biomarker in stomach adenocarcinoma**

Xingwei Wu^1,2+^，Shengnan Wang^3^，Chenci Wang^4^，Chengwei Wu^5^，Zhiyong Zhao^1*^

^1^Department of Thyroid and Breast Surgery, The Second Affiliated Hospital of Wannan Medical College, Wuhu 241000, Anhui, China;

^2^Clinical Laboratory, Traditional Chinese Hospital of Lu'an, Anhui University of Chinese Medicine Lu'an 237000, Anhui, China;

^3^Department of Pathology, Fuyang People's Hospital, Anhui Medical University,Fuyang 236000,Anhui,China;

^4^Department of Oncology, Funan County People’s Hospital,Fuyang 236000,Anhui,China;

^5^Department of Critical Care Medicine, The Second Hospital Affiliated to Jiaxing College,Jiaxing 314000,Zhejiang,China;

*Correspondence author: Zhiyong [Zhao, E-mail: silently00@sina.com](mailto:Zhao,silently00@sina.com)

+First author: Xingwei Wu

**Supplementary Figures and Figure Legends**

**
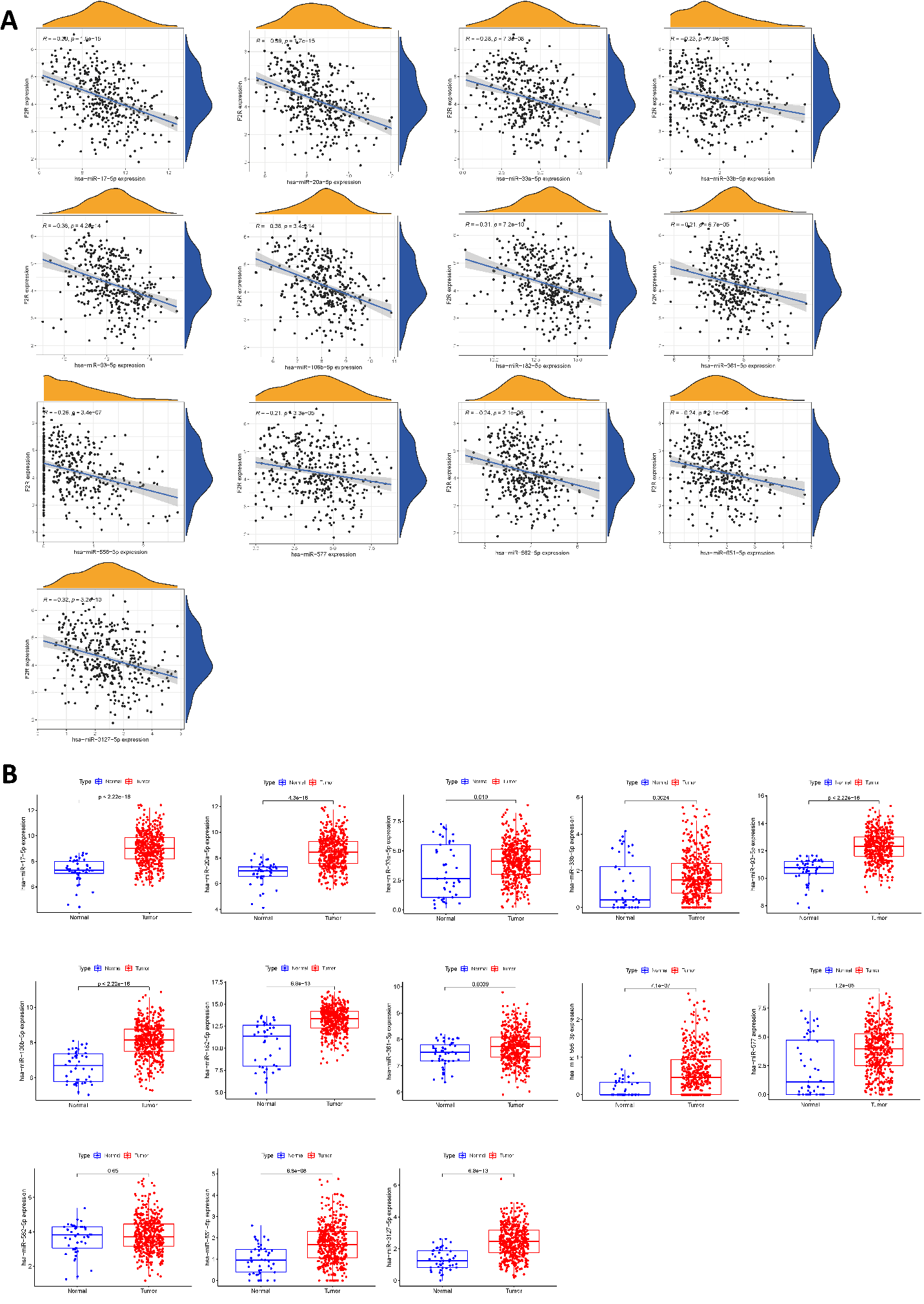
**

**Figure S1. The expression of miRNAs in STAD and the correlation between miRNAs and *F2R*.** (A) Correlation between miRNA and *F2R* in STAD. (B) Expression of miRNAs in STAD and related normal tissues based on the TCGA database.


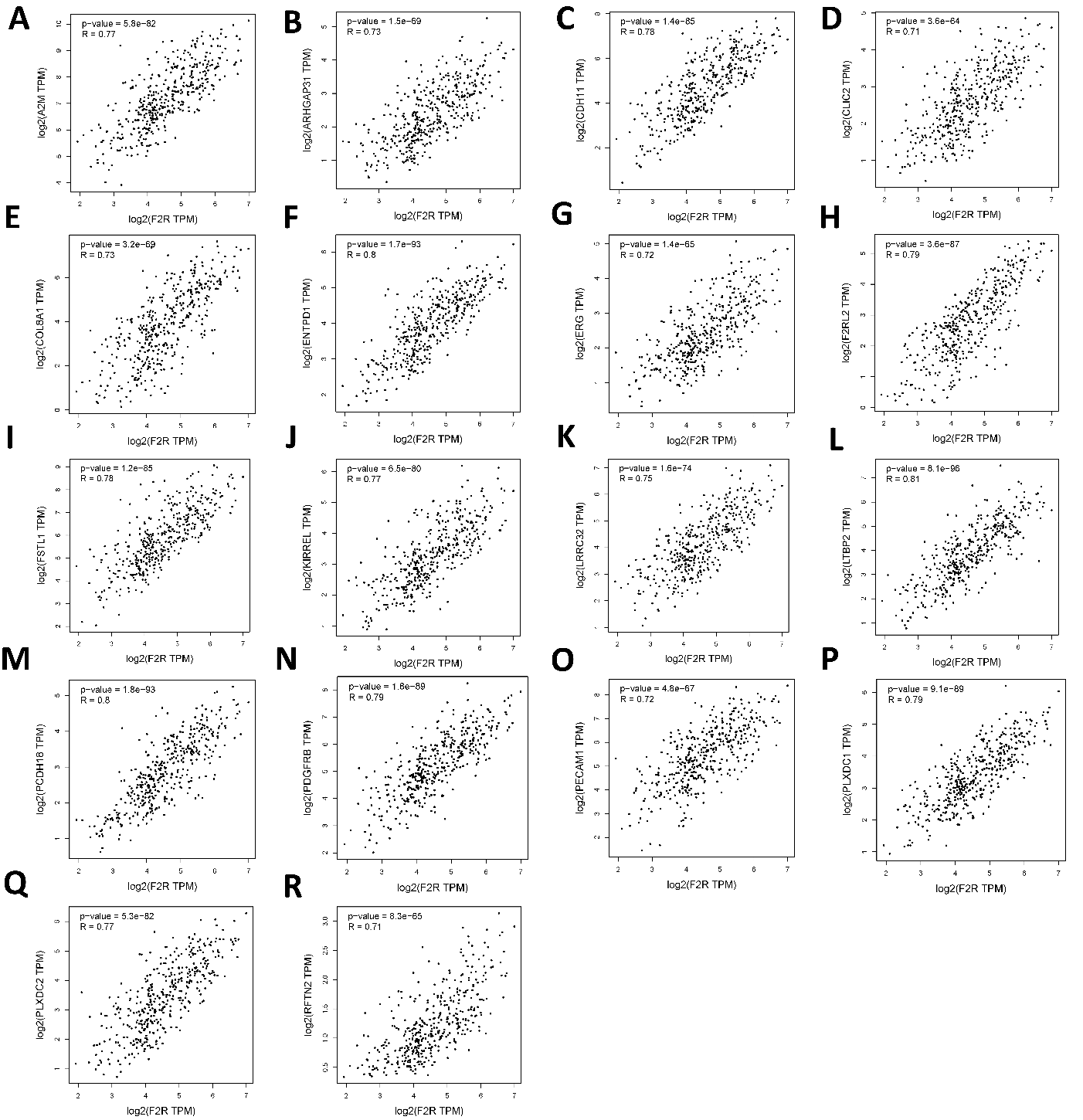


**Figure S2. Correlation of *F2R* with related differential genes.**


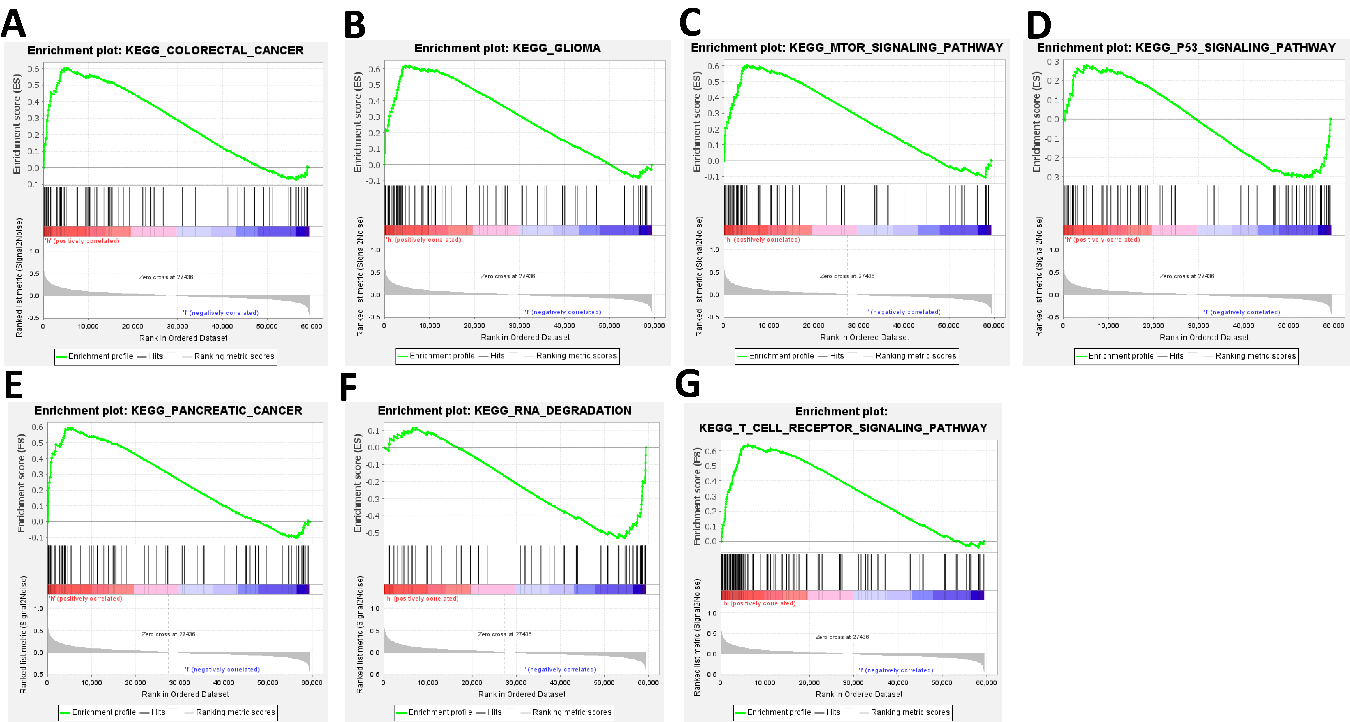


**Figure S3. GSEA analysis revealed that the *F2R* may be involved in biological functions.** (A); Relationship between *F2R* and colorectal cancer based on GSEA analysis (B); Glioma (C); mTOR signaling pathway (D); p53 signaling pathway (E); pancreatic cancer (F); RNA Degradation (G); T cell receptor signaling pathway.


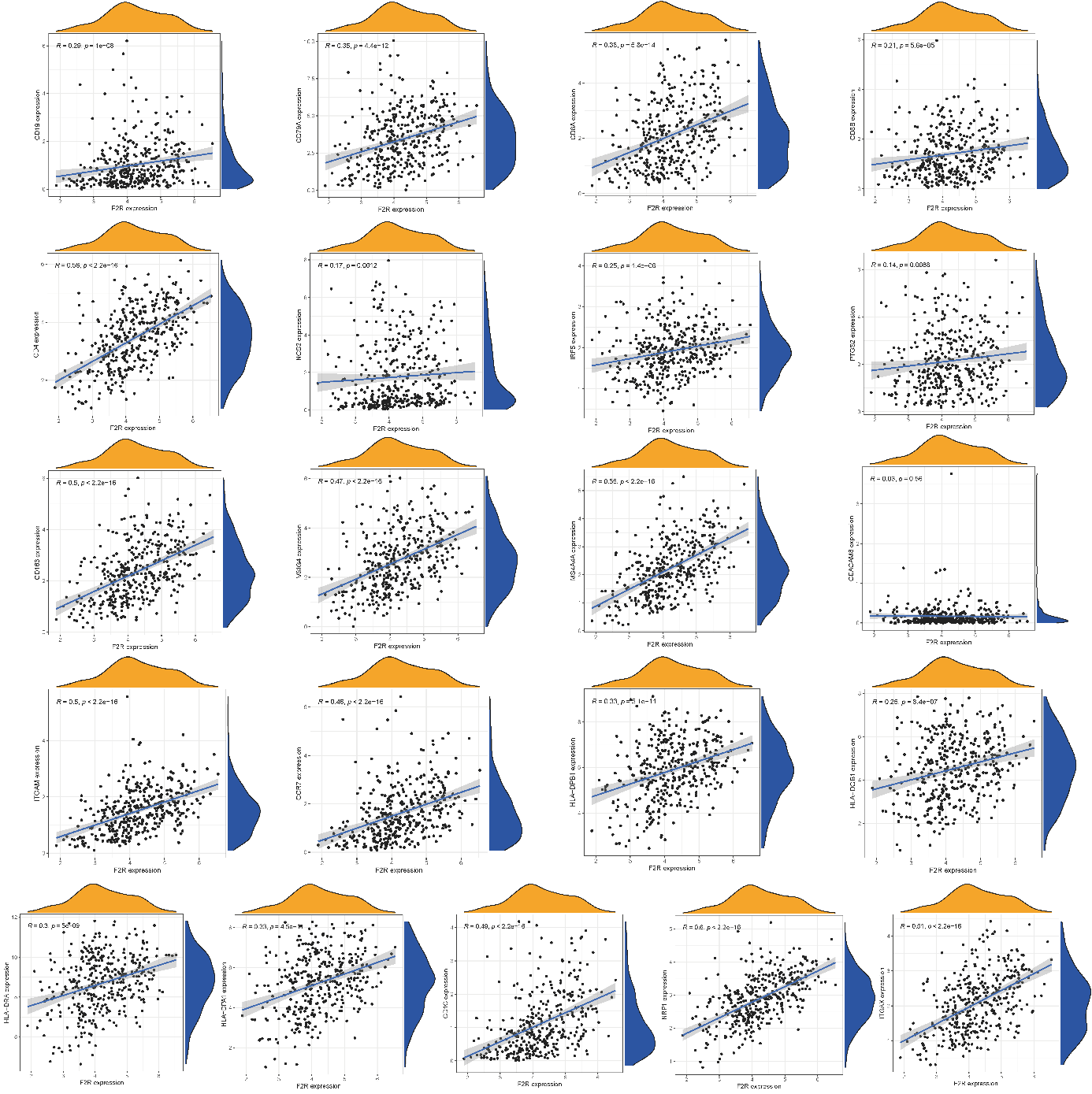


**Figure S4. Linear relationship between *F2R* and immune cell genes.**


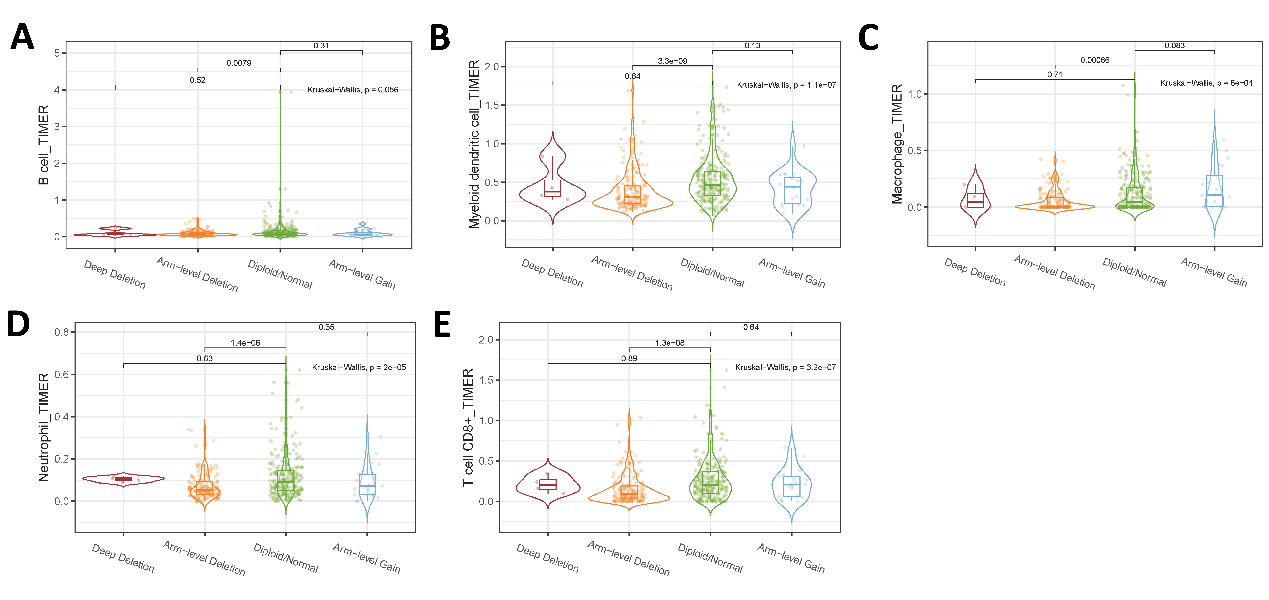


**Figure S5. The relationship between *F2R* gene copy number and immune cells.** (A) B cells based on the TIMER database. (B); Myeloid dendritic (C); Macrophage (D); Neutrophil (E); T cell CD8+.


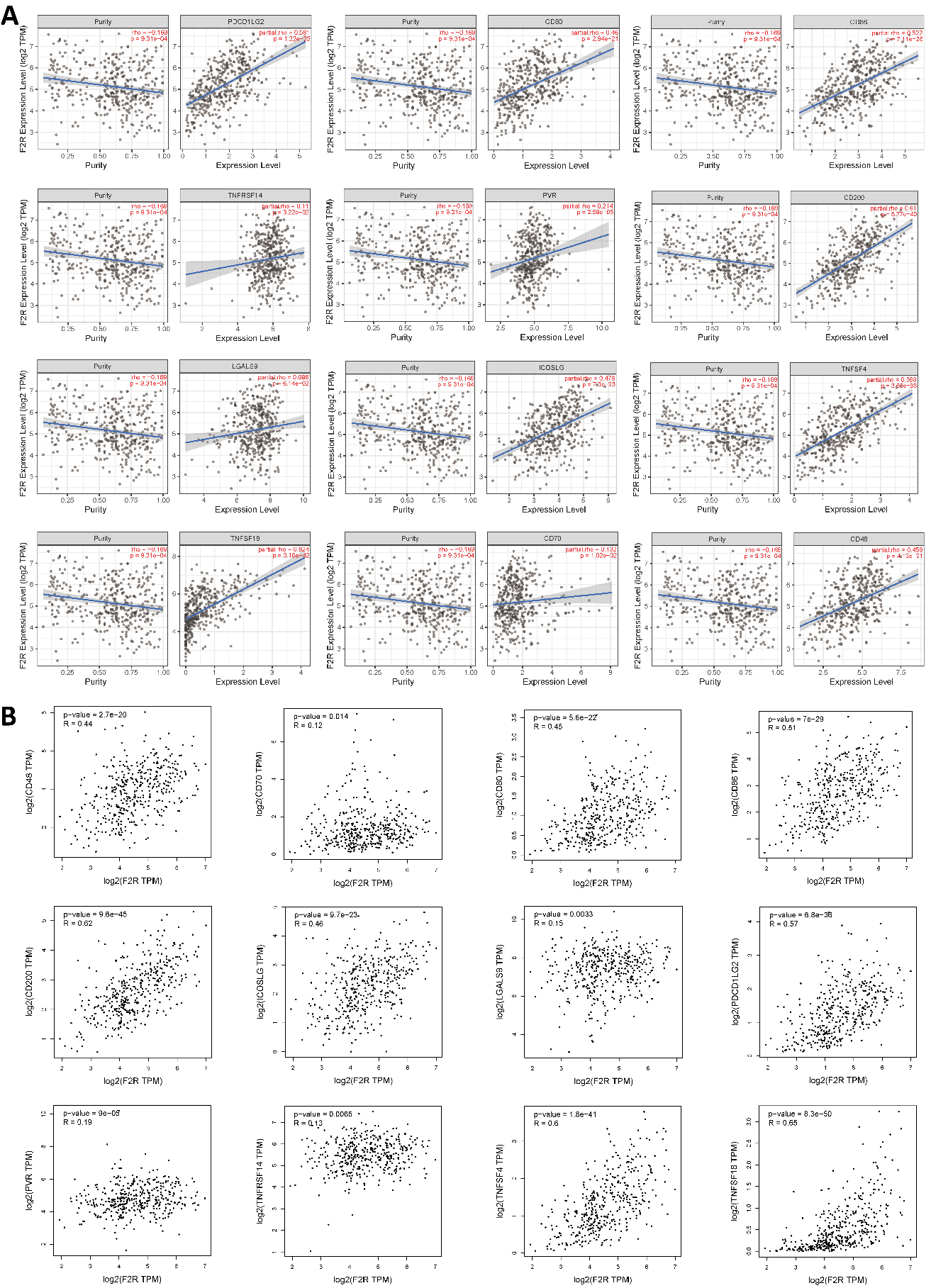


**Figure S6. Correlation between *F2R* and immune checkpoints.** (A) Correlation between *F2R* and immune checkpoints based on the TIMER database. (B) Correlation between *F2R* and immune checkpoints based on the GEPIA database.

**Table Legends**

**Table 1** STAD patient characteristics in the TCGA database. **(Excel.xlsx)**

**Table 2** *F2R* expression correlated with clinical pathological characteristics (logistic regression). **(Excel.xlsx)**

**Table 3** Univariate analysis and multivariate analyses of STAD patients overall survival in the TCGA database. **(Excel.xlsx)**

**Table S1** Clinical characteristics of STAD patients in the TCGA database. **(Excel.xlsx)**

**Table S2** Table of correlation between *F2R* and miRNA. **(Excel.xlsx)**

**Table S3** The correlation of *hsa-miR-144-5p* and lincRNA is shown in the table below. **(Excel.xlsx)**

**Table S4** The correlation of *hsa-miR-486-5p* and lincRNA is shown in the table below. **(Excel.xlsx)**

**Table S5** correlations between *F2R* and related genes. **(Excel.xlsx)**

**Table S6** GSEA results show biological functions associated with *F2R*. **(Excel.xlsx)**

**Table S7** Correlation of *F2R* with immune cell gene markers. **(Excel.xlsx)**

**Table S8** The methylation status of the *F2R* gene correlates with the prognosis of STAD patients. **(Excel.xlsx)**
