# Supplementary material for: Bioinformatics analysis identifies coagulation factor II receptor as a potential biomarker in stomach adenocarcinoma
Source: Sci Rep. 2024 Jan 30;14:2468. doi: 10.1038/s41598-024-52397-6 (PMC10827804; doi:10.1038/s41598-024-52397-6)
Supplement: Supplementary file 2 — Supplementary Table S1. [file 41598_2024_52397_MOESM2_ESM.docx]

**Table S1** Clinical characteristics of STAD patients in the TCGA database.

| Characteristic | levels | Overall |
| --- | --- | --- |
| n |  |  |
| Gender, n (%) | Female | 134(35.73%) |
|  | Male | 241(64.27%) |
| Age, n (%) | <=60 | 121(32.61%) |
|  | >60 | 250(67.39%) |
| Histologic grade, n (%) | G1 | 10(2.73%) |
|  | G2 | 137(37.43%) |
|  | G3 | 219(59.84%) |
| Pathologic stage, n (%) | Stage I | 53(15.06%) |
|  | Stage II | 111(31.53%) |
|  | Stage III | 150(42.61%) |
|  | Stage IV | 38(10.80%) |
| Tumor stage, n (%) | T1 | 19(5.18%) |
|  | T2 | 80(21.80%) |
|  | T3 | 168(45.78%) |
|  | T4 | 100(27.25%) |
| Node stage, n (%) | N0 | 111(31.09%) |
|  | N1 | 97(27.17%) |
|  | N2 | 75(21.01%) |
|  | N3 | 74(20.73%) |
| Metastasis stage, n (%) | M0 | 330(92.96%) |
|  | M1 | 25(7.04%) |
| Primary therapy outcome, n (%) | PD | 38(22.22%) |
|  | SD | 9(5.26%) |
|  | PR | 1(0.58%) |
|  | CR | 123(71.93%) |
| OS event, n (%) | Alive | 244(65.07%) |
|  | Dead | 131(34.93%) |
|  |  |  |
