# Supplementary material for: Bioinformatics analysis identifies coagulation factor II receptor as a potential biomarker in stomach adenocarcinoma
Source: Sci Rep. 2024 Jan 30;14:2468. doi: 10.1038/s41598-024-52397-6 (PMC10827804; doi:10.1038/s41598-024-52397-6)
Supplement: Supplementary file 3 — Supplementary Table S2. [file 41598_2024_52397_MOESM3_ESM.docx]

**Table S2** Table of correlation between F2R and miRNA.

| Gene | miRNA | cor | pvalue | logFC | diffPval |
| --- | --- | --- | --- | --- | --- |
| F2R | hsa-miR-17-5p | -0.39478 | 1.49E-15 | 1.744815 | 1.87E-16 |
| F2R | hsa-miR-20a-5p | -0.39426 | 1.74E-15 | 1.642563 | 4.31E-16 |
| F2R | hsa-miR-106b-5p | -0.38066 | 3.41E-14 | 1.499501 | 4.87E-17 |
| F2R | hsa-miR-93-5p | -0.37959 | 4.16E-14 | 1.703421 | 2.33E-18 |
| F2R | hsa-miR-3127-5p | -0.32042 | 3.16E-10 | 1.126431 | 6.78E-13 |
| F2R | hsa-miR-182-5p | -0.31417 | 7.16E-10 | 2.644755 | 6.78E-13 |
| F2R | hsa-miR-33a-5p | -0.27583 | 7.25E-08 | 0.755675 | 0.018898 |
| F2R | hsa-miR-556-3p | -0.26076 | 3.38E-07 | 0.414925 | 7.12E-07 |
| F2R | hsa-miR-582-5p | -0.24401 | 2.06E-06 | 0.175361 | 0.654862 |
| F2R | hsa-miR-651-5p | -0.24319 | 2.07E-06 | 0.753315 | 6.78E-08 |
| F2R | hsa-miR-33b-5p | -0.22939 | 7.85E-06 | 0.487437 | 0.002441 |
| F2R | hsa-miR-577 | -0.21346 | 3.31E-05 | 1.578221 | 1.21E-05 |
| F2R | hsa-miR-144-5p | -0.20911 | 5.00E-05 | -0.80793 | 0.000352 |
| F2R | hsa-miR-361-5p | -0.2057 | 6.65E-05 | 0.283559 | 0.003894 |
| F2R | hsa-miR-486-5p | -0.20074 | 9.99E-05 | -1.27222 | 8.77E-08 |
| F2R | hsa-miR-106a-5p | -0.18252 | 0.000411 | 0.544553 | 0.028612 |
| F2R | hsa-miR-190a-5p | -0.14013 | 0.006829 | 0.108305 | 0.463169 |
| F2R | hsa-miR-24-3p | -0.12262 | 0.018031 | 0.553786 | 1.32E-07 |
| F2R | hsa-miR-105-5p | -0.11134 | 0.031804 | 2.071211 | 0.006491 |
| F2R | hsa-miR-20b-5p | -0.07595 | 0.143646 | -0.43806 | 0.007928 |
| F2R | hsa-miR-181b-5p | -0.07062 | 0.173987 | 1.552465 | 2.14E-19 |
| F2R | hsa-miR-371a-3p | -0.06811 | 0.189948 | 0.184244 | 0.058003 |
| F2R | hsa-miR-489-3p | -0.04891 | 0.346863 | 0.184654 | 0.002893 |
| F2R | hsa-miR-130a-5p | -0.04587 | 0.377682 | 0.246954 | 0.003032 |
| F2R | hsa-miR-147a | -0.03938 | 0.448832 | ###### | 0.650078 |
| F2R | hsa-miR-491-5p | -0.03681 | 0.479112 | -0.22683 | 0.07801 |
| F2R | hsa-miR-1323 | -0.02863 | 0.582001 | 0.094949 | 0.858229 |
| F2R | hsa-miR-3924 | -0.01039 | 0.841628 | 0.002223 | 0.656554 |
| F2R | hsa-miR-181a-5p | -0.008 | 0.877771 | 1.446298 | 7.08E-19 |
| F2R | hsa-miR-181d-5p | -0.00648 | 0.900829 | 0.470672 | 0.000325 |
| F2R | hsa-miR-4731-5p | -0.00452 | 0.930813 | 0.008799 | 0.693855 |
| F2R | hsa-miR-588 | -0.00334 | 0.948735 | 0.009656 | 0.337873 |
| F2R | hsa-miR-520a-3p | -0.00226 | 0.965283 | 0.128121 | 0.829107 |
| F2R | hsa-miR-525-5p | -0.0009 | 0.986259 | 0.094744 | 0.524313 |
| F2R | hsa-miR-448 | 0.014562 | 0.779535 | 0.025006 | 0.229163 |
| F2R | hsa-miR-520a-5p | 0.014989 | 0.773239 | 0.14143 | 0.88248 |
| F2R | hsa-miR-542-3p | 0.02646 | 0.61079 | 1.385436 | 8.48E-15 |
| F2R | hsa-miR-450b-5p | 0.027584 | 0.595718 | 1.25467 | 2.14E-11 |
| F2R | hsa-miR-519d-3p | 0.036031 | 0.488413 | 0.092288 | 0.937012 |
| F2R | hsa-miR-494-3p | 0.041249 | 0.427632 | 0.536241 | 5.61E-06 |
| F2R | hsa-miR-5691 | 0.073373 | 0.157861 | -0.03471 | 0.012857 |
| F2R | hsa-miR-499b-5p | 0.122411 | 0.01818 | 0.014263 | 0.158921 |
| F2R | hsa-miR-328-3p | 0.125419 | 0.015551 | -0.42191 | 0.002473 |
| F2R | hsa-miR-23a-3p | 0.127094 | 0.014214 | 0.623551 | 1.43E-10 |
| F2R | hsa-miR-154-5p | 0.137524 | 0.007903 | 0.071534 | 0.426073 |
| F2R | hsa-miR-214-3p | 0.150377 | 0.003647 | 0.767402 | 3.50E-07 |
| F2R | hsa-miR-380-3p | 0.163182 | 0.001589 | 0.084414 | 0.149594 |
| F2R | hsa-miR-665 | 0.164966 | 0.001408 | -0.02027 | 0.305945 |
| F2R | hsa-miR-181c-5p | 0.167466 | 0.001203 | 0.324771 | 0.005083 |
| F2R | hsa-miR-541-3p | 0.19101 | 0.000211 | 0.294274 | 0.000143 |
| F2R | hsa-miR-23c | 0.193081 | 0.000179 | -0.07837 | 0.010336 |
| F2R | hsa-miR-23b-3p | 0.19503 | 0.000158 | -0.48664 | 4.09E-07 |
| F2R | hsa-miR-411-3p | 0.216315 | 2.58E-05 | 0.024521 | 0.918282 |
| F2R | hsa-miR-432-5p | 0.236373 | 4.31E-06 | 0.27847 | 0.026367 |
| F2R | hsa-miR-379-3p | 0.238567 | 3.27E-06 | -0.22482 | 0.004139 |
| F2R | hsa-miR-889-3p | 0.256628 | 5.75E-07 | 0.601145 | 5.33E-06 |
|  |  |  |  |  |  |
