# Supplementary material for: Bioinformatics analysis identifies coagulation factor II receptor as a potential biomarker in stomach adenocarcinoma
Source: Sci Rep. 2024 Jan 30;14:2468. doi: 10.1038/s41598-024-52397-6 (PMC10827804; doi:10.1038/s41598-024-52397-6)
Supplement: Supplementary file 4 — Supplementary Table S3. [file 41598_2024_52397_MOESM4_ESM.docx]

**Table S3** The correlation of hsa-miR-144-5p and lincRNA is shown in the table below.

| lncRNA | miRNA | cor | pvalue | logFC | diffPval |
| --- | --- | --- | --- | --- | --- |
| MAGI2-AS3 | hsa-miR-144-5p | -0.31704 | 4.93E-10 | -0.33386 | 0.018739 |
| LINC00294 | hsa-miR-144-5p | -0.27153 | 1.17E-07 | -0.15461 | 0.727587 |
| FTX | hsa-miR-144-5p | -0.21418 | 3.24E-05 | 0.142028 | 0.017813 |
| LINC01278 | hsa-miR-144-5p | -0.18878 | 0.000256 | -0.17763 | 0.787115 |
| LINC00963 | hsa-miR-144-5p | -0.18301 | 0.000396 | 0.199334 | 0.058912 |
| LINC00943 | hsa-miR-144-5p | -0.10143 | 0.050611 | 0.077364 | 6.57E-05 |
| NUTM2B-AS1 | hsa-miR-144-5p | -0.06562 | 0.206604 | 0.275687 | 1.50E-09 |
| LINC01126 | hsa-miR-144-5p | -0.04738 | 0.362036 | 0.191215 | 1.28E-07 |
| RBM26-AS1 | hsa-miR-144-5p | -0.04205 | 0.418572 | 0.097501 | 0.02422 |
| XIST | hsa-miR-144-5p | -0.03167 | 0.542539 | 0.125224 | 0.834414 |
| ANKRD10-IT1 | hsa-miR-144-5p | -0.01831 | 0.724675 | 1.374078 | 2.59E-14 |
| LINC00667 | hsa-miR-144-5p | 0.033352 | 0.521165 | -0.26114 | 0.00511 |
| DSCAM-AS1 | hsa-miR-144-5p | 0.053971 | 0.29917 | 0.04463 | 0.062329 |
| LINC02305 | hsa-miR-144-5p | 0.055066 | 0.289459 | 0.014567 | 0.086194 |
| GAS5 | hsa-miR-144-5p | 0.074229 | 0.152988 | 0.520898 | 6.83E-05 |
| MCM3AP-AS1 | hsa-miR-144-5p | 0.084505 | 0.103657 | 0.298592 | 1.99E-12 |
| NEAT1 | hsa-miR-144-5p | 0.197934 | 0.000125 | 0.642346 | 9.91E-05 |
|  |  |  |  |  |  |
