# Supplementary material for: Bioinformatics analysis identifies coagulation factor II receptor as a potential biomarker in stomach adenocarcinoma
Source: Sci Rep. 2024 Jan 30;14:2468. doi: 10.1038/s41598-024-52397-6 (PMC10827804; doi:10.1038/s41598-024-52397-6)
Supplement: Supplementary file 5 — Supplementary Table S4. [file 41598_2024_52397_MOESM5_ESM.docx]

**Table S4** The correlation of hsa-miR-486-5p and lincRNA is shown in the table below.

| lncRNA | miRNA | cor | pvalue | logFC | diffPval |
| --- | --- | --- | --- | --- | --- |
| PWAR5 | hsa-miR-486-5p | -0.22829 | 8.70E-06 | -0.50697 | 6.28E-07 |
| ZEB1-AS1 | hsa-miR-486-5p | -0.21684 | 2.57E-05 | 0.388874 | 5.52E-06 |
| SH3RF3-AS1 | hsa-miR-486-5p | -0.14257 | 0.005912 | 0.164483 | 0.209552 |
| LPP-AS2 | hsa-miR-486-5p | -0.14007 | 0.006854 | 0.072104 | 0.526558 |
| LINC00963 | hsa-miR-486-5p | -0.12355 | 0.017168 | 0.199334 | 0.058912 |
| DLGAP1-AS1 | hsa-miR-486-5p | -0.10244 | 0.048387 | 0.481881 | 0.000119 |
| MZF1-AS1 | hsa-miR-486-5p | -0.06028 | 0.246029 | 0.153447 | 0.001527 |
| PCAT7 | hsa-miR-486-5p | -0.04732 | 0.362561 | 0.593472 | 7.47E-06 |
| LINC01194 | hsa-miR-486-5p | -0.02946 | 0.571098 | 0.201395 | 0.000287 |
| PAUPAR | hsa-miR-486-5p | -0.02415 | 0.642376 | 0.009467 | 0.264102 |
| LINC00482 | hsa-miR-486-5p | 0.009675 | 0.852457 | 0.153454 | 0.0802 |
| SLC9A3-AS1 | hsa-miR-486-5p | 0.014192 | 0.784898 | 0.139566 | 0.904669 |
| LINC00174 | hsa-miR-486-5p | 0.031751 | 0.541376 | 0.666388 | 3.37E-09 |
| XIST | hsa-miR-486-5p | 0.052271 | 0.314675 | 0.125224 | 0.834414 |
| KCNQ1OT1 | hsa-miR-486-5p | 0.065122 | 0.210057 | 0.333111 | 1.32E-08 |
| NEAT1 | hsa-miR-486-5p | 0.163927 | 0.00153 | 0.642346 | 9.91E-05 |
|  |  |  |  |  |  |
