# Supplementary material for: Bioinformatics analysis identifies coagulation factor II receptor as a potential biomarker in stomach adenocarcinoma
Source: Sci Rep. 2024 Jan 30;14:2468. doi: 10.1038/s41598-024-52397-6 (PMC10827804; doi:10.1038/s41598-024-52397-6)
Supplement: Supplementary file 6 — Supplementary Table S5. [file 41598_2024_52397_MOESM6_ESM.docx]

**Table S5** correlations between F2R and related genes.

| Query | Gene | cor | pvalue |
| --- | --- | --- | --- |
| F2R | PCDH18 | 0.78506 | 1.35E-79 |
| F2R | LRRC8C | 0.601364 | 3.02E-38 |
| F2R | LDB2 | 0.601423 | 2.96E-38 |
| F2R | NID2 | 0.657522 | 8.6E-48 |
| F2R | ACVRL1 | 0.653375 | 5.12E-47 |
| F2R | EDNRA | 0.633383 | 1.9E-43 |
| F2R | RECK | 0.659513 | 3.62E-48 |
| F2R | FLI1 | 0.603652 | 1.34E-38 |
| F2R | CSGALNACT2 | 0.673591 | 6.51E-51 |
| F2R | BICC1 | 0.661492 | 1.52E-48 |
| F2R | PLXDC1 | 0.731705 | 4.59E-64 |
| F2R | PECAM1 | 0.676777 | 1.48E-51 |
| F2R | LAMA2 | 0.686241 | 1.64E-53 |
| F2R | MMRN2 | 0.629661 | 8.22E-43 |
| F2R | TCF21 | 0.62286 | 1.13E-41 |
| F2R | GIMAP6 | 0.639909 | 1.39E-44 |
| F2R | EDIL3 | 0.612312 | 5.86E-40 |
| F2R | HIC1 | 0.655537 | 2.03E-47 |
| F2R | CSF1R | 0.617044 | 1.02E-40 |
| F2R | AP001189.3 | 0.747737 | 2.56E-68 |
| F2R | SYT11 | 0.616804 | 1.11E-40 |
| F2R | IGFBP5 | 0.621521 | 1.89E-41 |
| F2R | FBXL7 | 0.62156 | 1.86E-41 |
| F2R | GGT5 | 0.654631 | 2.99E-47 |
| F2R | LTBP2 | 0.776726 | 6.88E-77 |
| F2R | STARD8 | 0.63254 | 2.65E-43 |
| F2R | SYDE1 | 0.62352 | 8.82E-42 |
| F2R | COL14A1 | 0.639567 | 1.6E-44 |
| F2R | ELN | 0.633747 | 1.65E-43 |
| F2R | ZEB2 | 0.675399 | 2.82E-51 |
| F2R | ERG | 0.675556 | 2.62E-51 |
| F2R | ANTXR1 | 0.651085 | 1.35E-46 |
| F2R | ECM2 | 0.653429 | 5E-47 |
| F2R | LHFPL2 | 0.601889 | 2.51E-38 |
| F2R | MYCT1 | 0.604556 | 9.71E-39 |
| F2R | LRRC32 | 0.770635 | 5.53E-75 |
| F2R | FRZB | 0.604743 | 9.09E-39 |
| F2R | CYYR1 | 0.607969 | 2.85E-39 |
| F2R | CTSK | 0.658521 | 5.57E-48 |
| F2R | CALCRL | 0.620112 | 3.22E-41 |
| F2R | NID1 | 0.645093 | 1.66E-45 |
| F2R | CD34 | 0.609241 | 1.8E-39 |
| F2R | PTPRM | 0.631602 | 3.84E-43 |
| F2R | TMEM119 | 0.64714 | 7.1E-46 |
| F2R | DIO2 | 0.712632 | 2.18E-59 |
| F2R | PDGFRB | 0.758718 | 2.02E-71 |
| F2R | PDGFRA | 0.678798 | 5.74E-52 |
| F2R | HMCN1 | 0.656904 | 1.12E-47 |
| F2R | ARHGEF6 | 0.645546 | 1.38E-45 |
| F2R | PLXNC1 | 0.671553 | 1.66E-50 |
| F2R | DCHS1 | 0.61169 | 7.36E-40 |
| F2R | PLXDC2 | 0.751892 | 1.79E-69 |
| F2R | ATP8B2 | 0.621751 | 1.73E-41 |
| F2R | LAMA4 | 0.70955 | 1.15E-58 |
| F2R | GASK1B | 0.603402 | 1.47E-38 |
| F2R | VASH1 | 0.629764 | 7.9E-43 |
| F2R | COL8A1 | 0.685562 | 2.27E-53 |
| F2R | F2RL2 | 0.772569 | 1.39E-75 |
| F2R | COL6A3 | 0.635783 | 7.32E-44 |
| F2R | CYGB | 0.689225 | 3.81E-54 |
| F2R | HACD4 | 0.607759 | 3.08E-39 |
| F2R | C1S | 0.683102 | 7.43E-53 |
| F2R | S1PR1 | 0.633286 | 1.98E-43 |
| F2R | FMNL3 | 0.668182 | 7.69E-50 |
| F2R | OLFML1 | 0.697336 | 6.64E-56 |
| F2R | GIMAP8 | 0.627165 | 2.17E-42 |
| F2R | JAM3 | 0.628841 | 1.13E-42 |
| F2R | SLC24A3 | 0.604289 | 1.07E-38 |
| F2R | WIPF1 | 0.609469 | 1.66E-39 |
| F2R | VSTM4 | 0.62749 | 1.91E-42 |
| F2R | GLI1 | 0.67333 | 7.34E-51 |
| F2R | ARSB | 0.659408 | 3.79E-48 |
| F2R | PODN | 0.68209 | 1.21E-52 |
| F2R | A2M | 0.760762 | 5.11E-72 |
| F2R | STAB1 | 0.665942 | 2.11E-49 |
| F2R | DCN | 0.655281 | 2.26E-47 |
| F2R | CLIC2 | 0.653808 | 4.26E-47 |
| F2R | RFTN1 | 0.689419 | 3.47E-54 |
| F2R | GUCY1A1 | 0.679385 | 4.35E-52 |
| F2R | IGFBP7 | 0.663132 | 7.36E-49 |
| F2R | ADGRA2 | 0.72255 | 9.03E-62 |
| F2R | FBLN5 | 0.639134 | 1.9E-44 |
| F2R | GUCY1B1 | 0.656998 | 1.08E-47 |
| F2R | FSTL1 | 0.705466 | 9.97E-58 |
| F2R | DAB2 | 0.622761 | 1.18E-41 |
| F2R | KIAA1755 | 0.688301 | 5.99E-54 |
| F2R | HEYL | 0.70107 | 9.82E-57 |
| F2R | PLEKHO2 | 0.621995 | 1.58E-41 |
| F2R | FZD4 | 0.62584 | 3.62E-42 |
| F2R | MOXD1 | 0.654724 | 2.87E-47 |
| F2R | LTBP1 | 0.603739 | 1.3E-38 |
| F2R | VCAN | 0.680386 | 2.71E-52 |
| F2R | C1R | 0.637104 | 4.31E-44 |
| F2R | DKK3 | 0.608582 | 2.28E-39 |
| F2R | GPC6 | 0.684527 | 3.75E-53 |
| F2R | KIRREL1 | 0.707725 | 3.03E-58 |
| F2R | TCF4 | 0.673877 | 5.7E-51 |
| F2R | PALM2AKAP2 | 0.621246 | 2.09E-41 |
| F2R | ITGBL1 | 0.682491 | 9.96E-53 |
| F2R | FBN1 | 0.717761 | 1.32E-60 |
| F2R | EFEMP1 | 0.654382 | 3.33E-47 |
| F2R | TEK | 0.67768 | 9.71E-52 |
| F2R | CRISPLD2 | 0.636831 | 4.81E-44 |
| F2R | OLFML2B | 0.619983 | 3.38E-41 |
| F2R | GPR34 | 0.642932 | 4.05E-45 |
| F2R | RASSF2 | 0.616691 | 1.16E-40 |
| F2R | LZTS1 | 0.643044 | 3.86E-45 |
| F2R | CDH11 | 0.71801 | 1.15E-60 |
| F2R | LSAMP | 0.631246 | 4.42E-43 |
| F2R | TLR7 | 0.605949 | 5.9E-39 |
| F2R | RASL12 | 0.611695 | 7.35E-40 |
| F2R | AEBP1 | 0.602612 | 1.94E-38 |
| F2R | FAT4 | 0.655124 | 2.42E-47 |
| F2R | CD93 | 0.643151 | 3.7E-45 |
| F2R | ITGA4 | 0.652909 | 6.25E-47 |
| F2R | LUM | 0.669363 | 4.5E-50 |
| F2R | ST8SIA4 | 0.606406 | 5.01E-39 |
| F2R | PREX2 | 0.636287 | 5.98E-44 |
| F2R | CYBRD1 | 0.614056 | 3.09E-40 |
| F2R | ARHGAP31 | 0.693043 | 5.76E-55 |
| F2R | DLC1 | 0.627452 | 1.94E-42 |
| F2R | CDH5 | 0.612995 | 4.56E-40 |
| F2R | SH2B3 | 0.605392 | 7.2E-39 |
| F2R | ENTPD1 | 0.713303 | 1.52E-59 |
| F2R | IL1R1 | 0.634463 | 1.24E-43 |
| F2R | GAS7 | 0.690657 | 1.88E-54 |
| F2R | ZNF521 | 0.658981 | 4.56E-48 |
| F2R | ETS1 | 0.620443 | 2.84E-41 |
| F2R | GNB4 | 0.63968 | 1.52E-44 |
| F2R | PRR16 | 0.604249 | 1.08E-38 |
| F2R | PDE1A | 0.667683 | 9.63E-50 |
| F2R | HEG1 | 0.739446 | 4.44E-66 |
|  |  |  |  |
