# Supplementary material for: Bioinformatics analysis identifies coagulation factor II receptor as a potential biomarker in stomach adenocarcinoma
Source: Sci Rep. 2024 Jan 30;14:2468. doi: 10.1038/s41598-024-52397-6 (PMC10827804; doi:10.1038/s41598-024-52397-6)
Supplement: Supplementary file 7 — Supplementary Table S6. [file 41598_2024_52397_MOESM7_ESM.docx]

**Table S6** GSEA results show biological functions associated with F2R.

| NAME | ES | NES | NOM p-val | FDR q-val |
| --- | --- | --- | --- | --- |
| KEGG_JAK_STAT_SIGNALING_PATHWAY | 0.646322 | 2.437007 | 0 | 0 |
| KEGG_TOLL_LIKE_RECEPTOR_SIGNALING_PATHWAY | 0.63363 | 2.239335 | 0 | 8.97E-05 |
| KEGG_HEDGEHOG_SIGNALING_PATHWAY | 0.695084 | 2.221374 | 0 | 1.71E-04 |
| KEGG_CHEMOKINE_SIGNALING_PATHWAY | 0.645003 | 2.208449 | 0 | 1.55E-04 |
| KEGG_MAPK_SIGNALING_PATHWAY | 0.57766 | 2.187685 | 0 | 2.26E-04 |
| KEGG_CALCIUM_SIGNALING_PATHWAY | 0.581195 | 2.183312 | 0 | 1.99E-04 |
| KEGG_TGF_BETA_SIGNALING_PATHWAY | 0.645008 | 2.090863 | 0 | 9.57E-04 |
| KEGG_NEUROTROPHIN_SIGNALING_PATHWAY | 0.610509 | 2.053283 | 0 | 0.0017178 |
| KEGG_T_CELL_RECEPTOR_SIGNALING_PATHWAY | 0.640282 | 2.04911 | 0.003846154 | 0.0016745 |
| KEGG_ADIPOCYTOKINE_SIGNALING_PATHWAY | 0.61145 | 2.048729 | 0 | 0.0016186 |
| KEGG_MTOR_SIGNALING_PATHWAY | 0.604654 | 1.929887 | 0.001976285 | 0.0063999 |
| KEGG_FC_EPSILON_RI_SIGNALING_PATHWAY | 0.542664 | 1.859633 | 0.00814664 | 0.011338 |
| KEGG_VEGF_SIGNALING_PATHWAY | 0.542738 | 1.854267 | 0.004032258 | 0.01155 |
| KEGG_ERBB_SIGNALING_PATHWAY | 0.563577 | 1.849746 | 0.0078125 | 0.0118838 |
| KEGG_B_CELL_RECEPTOR_SIGNALING_PATHWAY | 0.599145 | 1.819959 | 0.023762377 | 0.0142503 |
| KEGG_GNRH_SIGNALING_PATHWAY | 0.503397 | 1.813421 | 0.003921569 | 0.0146933 |
| KEGG_INSULIN_SIGNALING_PATHWAY | 0.507979 | 1.80314 | 0.006122449 | 0.0157122 |
| KEGG_RIG_I_LIKE_RECEPTOR_SIGNALING_PATHWAY | 0.513043 | 1.793876 | 0.006237006 | 0.0164397 |
| KEGG_NOD_LIKE_RECEPTOR_SIGNALING_PATHWAY | 0.575981 | 1.793591 | 0.004106776 | 0.0162587 |
| KEGG_WNT_SIGNALING_PATHWAY | 0.502963 | 1.774105 | 0.005882353 | 0.0182562 |
|  |  |  |  |  |

Gene sets with an NOM p value < 0.05 and an FDR q-value < 0.25 were considered to be significant. ES, enrichment score; NES, normalized enrichment score; NOM, nominal; and FDR, false discovery rate
