# Supplementary material for: Bioinformatics analysis identifies coagulation factor II receptor as a potential biomarker in stomach adenocarcinoma
Source: Sci Rep. 2024 Jan 30;14:2468. doi: 10.1038/s41598-024-52397-6 (PMC10827804; doi:10.1038/s41598-024-52397-6)
Supplement: Supplementary file 8 — Supplementary Table S7. [file 41598_2024_52397_MOESM8_ESM.docx]

**Table S7** Correlation of F2R with immune cell gene markers.

| immuneCell | gene | cor | pvalue |
| --- | --- | --- | --- |
| B cell | CD19 | 0.291549 | 1.03E-08 |
| B cell | CD79A | 0.349579 | 4.39E-12 |
| CD8+ T cell | CD8A | 0.37636 | 5.82E-14 |
| CD8+ T cell | CD8B | 0.206595 | 5.76E-05 |
| CD4+ T cell | CD4 | 0.557956 | 0 |
| M1 macrophage | NOS2 | 0.166718 | 0.00121 |
| M1 macrophage | IRF5 | 0.246957 | 1.39E-06 |
| M1 macrophage | PTGS2 | 0.135121 | 0.008838 |
| M2 macrophage | CD163 | 0.501181 | 0 |
| M2 macrophage | VSIG4 | 0.470002 | 0 |
| M2 macrophage | MS4A4A | 0.548363 | 0 |
| Neutrophil | CEACAM8 | 0.03006 | 0.561713 |
| Neutrophil | ITGAM | 0.500536 | 0 |
| Neutrophil | CCR7 | 0.461165 | 0 |
| Dendritic cell | HLA-DPB1 | 0.33252 | 5.15E-11 |
| Dendritic cell | HLA-DQB1 | 0.251923 | 8.42E-07 |
| Dendritic cell | HLA-DRA | 0.297464 | 5.04E-09 |
| Dendritic cell | HLA-DPA1 | 0.33356 | 4.45E-11 |
| Dendritic cell | CD1C | 0.490597 | 0 |
| Dendritic cell | NRP1 | 0.595749 | 0 |
| Dendritic cell | ITGAX | 0.509868 | 0 |
|  |  |  |  |
