# Supplementary material for: Bioinformatics analysis identifies coagulation factor II receptor as a potential biomarker in stomach adenocarcinoma
Source: Sci Rep. 2024 Jan 30;14:2468. doi: 10.1038/s41598-024-52397-6 (PMC10827804; doi:10.1038/s41598-024-52397-6)
Supplement: Supplementary file 9 — Supplementary Table S8. [file 41598_2024_52397_MOESM9_ESM.docx]

**Table S8** The methylation status of the F2R gene correlates with the prognosis of STAD patients.

| CpG island | HR | p-value |
| --- | --- | --- |
| **TSS200-Island-cg02047489** | **0.715** | **0.048** |
| TSS200-Island-cg06994090 | 0.859 | 0.44 |
| **TSS200-Island-cg11627632** | **0.695** | **0.031** |
| **Body-Island-cg03666316** | **0.521** | **0.0011** |
| **TSS1500-Island-cg04528371** | **0.621** | **0.025** |
| **TSS1500-Island-cg13436055** | **0.504** | **0.0022** |
| TSS1500-Island-cg14718568 | 0.699 | 0.08 |
| **TSS1500-Island-cg14773260** | **0.626** | **0.026** |
| **TSS1500-Island-cg20030243** | **0.648** | **0.032** |
| **TSS1500-Island-cg24678700** | **0.666** | **0.021** |
| **TSS1500-N_shore-cg03043127** | **0.699** | **0.03** |
| TSS1500-N_shore-cg14714391 | 0.715 | 0.11 |
| **TSS1500-N_shore-cg19735421** | **0.621** | **0.02** |
| **TSS1500-N_shore-cg26594335** | **0.677** | **0.026** |
| **Body-S_shore-cg11591325** | **0.649** | **0.014** |
| Body-S_shore-cg26997028 | 1.433 | 0.053 |
| Body-Open-Sea-cg12524168 | 0.73 | 0.12 |
| **Body-Open-Sea-cg20417124** | **0.662** | **0.044** |
| **Body-S-shelf-cg24702798** | **0.686** | **0.021** |
|  |  |  |
